# Conserved regulation of neurodevelopmental processes and behavior by FoxP in *Drosophila*

**DOI:** 10.1371/journal.pone.0211652

**Published:** 2019-02-12

**Authors:** Anna Castells-Nobau, Ilse Eidhof, Michaela Fenckova, Dova B. Brenman-Suttner, Jolanda M. Scheffer-de Gooyert, Sheren Christine, Rosa L. Schellevis, Kiran van der Laan, Christine Quentin, Lisa van Ninhuijs, Falko Hofmann, Radoslaw Ejsmont, Simon E. Fisher, Jamie M. Kramer, Stephan J. Sigrist, Anne F. Simon, Annette Schenck

**Affiliations:** 1 Department of Human Genetics, Donders Institute for Brain, Cognition and Behaviour, Radboud University Medical Center, Nijmegen, the Netherlands; 2 Department of Biology, Faculty of Science, Western University, London, Ontario, Canada; 3 Genetics, Institute of Biology, Freie Universität Berlin, Berlin, Germany; 4 NeuroCure Cluster of Excellence, Charité Universitätsmedizin Berlin, Berlin, Germany; 5 Max Planck Institute of Molecular Cell Biology and Genetics (MPI-CBG), Dresden, Germany; 6 Language and Genetics Department, Max Planck Institute of Psycholinguistics, Nijmegen, The Netherlands; 7 Donders Institute for Brain, Cognition and Behaviour, Radboud University, Nijmegen, the Netherlands; National Centre for Biological Sciences, TIFR, INDIA

## Abstract

FOXP proteins form a subfamily of evolutionarily conserved transcription factors involved in the development and functioning of several tissues, including the central nervous system. In humans, mutations in FOXP1 and FOXP2 have been implicated in cognitive deficits including intellectual disability and speech disorders. *Drosophila* exhibits a single ortholog, called FoxP, but due to a lack of characterized mutants, our understanding of the gene remains poor. Here we show that the dimerization property required for mammalian FOXP function is conserved in *Drosophila*. In flies, FoxP is enriched in the adult brain, showing strong expression in ~1000 neurons of cholinergic, glutamatergic and GABAergic nature. We generate *Drosophila* loss-of-function mutants and *UAS-FoxP* transgenic lines for ectopic expression, and use them to characterize FoxP function in the nervous system. At the cellular level, we demonstrate that *Drosophila* FoxP is required in larvae for synaptic morphogenesis at axonal terminals of the neuromuscular junction and for dendrite development of dorsal multidendritic sensory neurons. In the developing brain, we find that FoxP plays important roles in α-lobe mushroom body formation. Finally, at a behavioral level, we show that *Drosophila* FoxP is important for locomotion, habituation learning and social space behavior of adult flies. Our work shows that *Drosophila* FoxP is important for regulating several neurodevelopmental processes and behaviors that are related to human disease or vertebrate disease model phenotypes. This suggests a high degree of functional conservation with vertebrate FOXP orthologues and established flies as a model system for understanding FOXP related pathologies.

## Introduction

The forkhead box P (FOXP) transcription factors form a subfamily of evolutionarily conserved proteins. In mammals, the subfamily consists of four members, FOXP1-4, which have a wide range of important biological functions. FOXP1, FOXP2 and FOXP4 are highly homologous, present partially overlapping expression patterns in vertebrate brains [1, 2] and are involved, amongst other tissues, in the development and functioning of the central nervous system (CNS) [3]. FOXP3, evolutionarily the most distal member of the subfamily, is known for its expression and function in the immune system [4].

Rare mutations disrupting the human *FOXP1* and *FOXP2* genes cause distinct but overlapping neurodevelopmental and neuropsychiatric disorders [5]. Heterozygous *FOXP1* mutations lead to intellectual disability, speech deficits and autism spectrum disorder (ASD), and delayed motor development [6, 7]. Heterozygous *FOXP2* mutations cause childhood apraxia of speech (also known as developmental verbal dyspraxia), a severe speech disorder [8], and most of the affected individuals also show mild cognitive impairments, which are most apparent for the verbal domain [9, 10]. Genome-wide association studies recently reported that common intronic polymorphisms of *FOXP2* are associated with Attention-Deficit/Hyperactivity Disorder (ADHD) risk [11]. Functional studies in model organisms have extended our knowledge on FOXP protein functions in the CNS. Studies in mouse models revealed that Foxp1 helps direct motor axon projections to their peripheral targets during spinal cord development [12, 13] and is involved in dendritic morphogenesis of striatal neurons [14]. Ablation of Foxp1 in mouse brains leads to decreased social interest and higher occurrence of repetitive motor behaviors [14], suggesting conserved roles in behaviors that are relevant to ASD. Many studies have emphasized the importance of FOXP2 in the CNS, reporting roles in neurogenesis [15], neurite outgrowth [16, 17], dendrite morphogenesis [18, 19], and synaptic plasticity [20, 21]. In line with its involvement in human vocal behaviors, some Foxp2 mouse mutants are reported to show defects in pup and adult ultrasonic vocalizations [22], and studies of the avian ortholog in zebra finches (*Taeniopygia guttata*) revealed key contributions to song learning [23]. Foxp2 has been linked to other behaviors in mice, including social conduct [24], motor-skill learning and motor-control [18, 21, 25]. FOXP4 is expressed in several brain regions in vertebrates [26–29]. Although FOXP4 functions have not been studied extensively, it has been suggested to be involved in midbrain and hindbrain patterning, and to be essential for maintaining dendritic arborization of Purkinje cells and their association with glial fibers [30]. In summary, the *FOXP* gene subfamily plays critical roles in multiple neurodevelopmental processes including neuron morphogenesis and brain development, as well as behaviors such as skill learning, vocal communication, social and fine motor behaviors.

**Fig 1:**
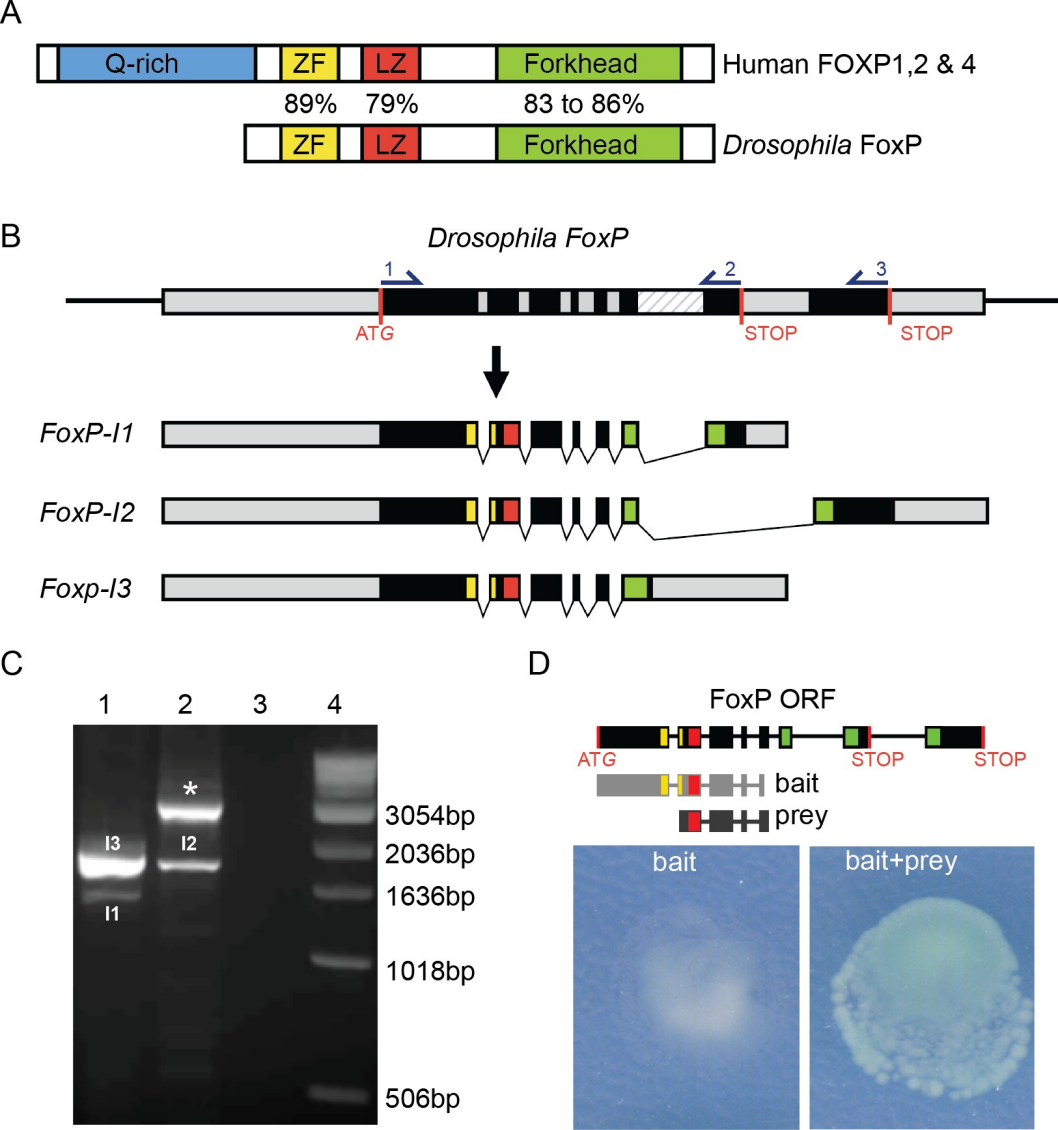
Conserved properties of *Drosophila FoxP*. (A) Schematic representation of human FOXP and *Drosophila* FoxP proteins. FOXP protein domains: glutamate-rich region (Q-rich, in blue), zinc finger (ZF, in yellow), leucine zipper (LZ, in red) and forkhead domain (Forkhead, in green) are indicated. % indicates aa similarity between human FOXP1, 2, 4 and *Drosophila* FoxP protein domains. (B) The *Drosophila FoxP* genomic region (exons in black, untranslated regions (UTRs) and introns in grey, and intron 6 who’s retention gives rise to FoxP-I3 in a striped pattern, START and alternative STOP codons in red) and the three encoded transcripts (FoxP-I1 to–I3). Protein domain-encoding regions are highlighted using the color code used in (A). Primers for RT-PCR analysis are indicated with numbers in the *FoxP* genomic region. (C) Agarose gel analysis of RT-PCR products amplified with primers 1–2 in lane 1 (PCR products corresponding to *FoxP-I1* (I1, 1329bp) and -*I3* (I3, 1701bp)) and primers 1–3 in lane 2 (lower band corresponds to *FoxP-I2* (I2, 2999bp), upper band (*) corresponds to an amplicon derived either from an unspliced *FoxP* pre-mRNA or amplification of genomic DNA present in the sample (2824bp)). Lane 3: negative control (primers, no template). Lane 4: molecular weight marker. (D) FoxP-FoxP dimerization in the yeast two-hybrid assay. The utilized construct (light grey) and isolated FoxP fragment (prey, dark grey) are depicted. The yeast two-hybrid bait alone shows no autoactivation and growth. When yeast are co-transformed with both, bait and prey induce colony growth and β-galactosidase activity, demonstrating FoxP dimerization.

The *FoxP* gene first arose in eukaryotic unicellular organisms, and multiple gene duplications led to the appearance of the four different members of the subfamily in vertebrates. Invertebrates, however, contain a sole copy of the *FoxP* gene in their genome [2]. The *Drosophila FoxP* gene is composed of 8 exons and can be transcribed into three isoforms by alternative splicing (Fig 1B) [2, 31] *FoxP Isoform-1* (*FoxP-I1*) and *FoxP-Isoform* 2 (*FoxP-I2*) differ in the inclusion of an alternative last exon, which leads to the transcription of a partially distinct forkhead domain that has different DNA binding sites [2]. A poorly described third isoform, *FoxP Isoform-3* (*FoxP-I3*) is characterized by retention of intron 6 [2, 31]. *FoxP-I3* is predicted to code for a protein with a truncated forkhead domain due to a premature stop codon located in the retained intronic region, resembling isoforms that lack the forkhead domain described also in humans [32].

*Drosophila* FoxP and human FOXP1/2/4 show a high degree of conservation in several functional protein domains, in particular in the zinc finger/leucine zipper region and the forkhead protein domains, involved in dimerization and DNA binding respectively. Nonetheless, *Drosophila* FoxP lacks the N-terminal Poly-Q stretches that might have transcriptional regulatory properties in mammals [33]. Mammalian FOXP proteins form dimers, required for DNA binding [34, 35]. *Drosophila* FoxP has also been reported to dimerize with itself in two genome-scale protein interaction screens [36, 37], but has not been confirmed. The high degree of FoxP conservation in a highly genetically tractable organism such as *Drosophila melanogaster* provides many possibilities for advancing our understanding of this gene in CNS development and function.

Through manipulation of *FoxP* promoter elements, several studies reported FoxP expression in the CNS, but the described patterns have been notably inconsistent between reports, ranging from wide expression of *Drosophila* FoxP in several regions of the CNS including the protocerebral bridge [38], to expression restricted to the αβ core and γ lobes of the mushroom bodies [39, 40]. Investigation using an anti-FoxP antibody also suggested a wide distribution of FoxP expressing cells within the CNS [38].

At the behavioral level FoxP is important for olfactory discrimination, influencing speed and accuracy of perceptual decision-making upon presentation of different odors [39, 41]. FoxP regulates this process by controlling the abundance of the voltage-gated potassium channel *shal*, which determines the spike threshold of the αβ core Kenyon cells in the mushroom body [41]. FoxP has also been reported to play roles in motor coordination, courtship behavior, courtship song generation [38], and is required for operant self-learning, a form of motor learning [42]. These studies suggest that *Drosophila* FoxP regulates a broad range of behaviors. However, all these studies employed RNA interference (RNAi) against *FoxP* mRNA or uncharacterized P-element insertions in the *FoxP* gene, which (based on the location of the insertion locus) are likely to affect only the *FoxP-I2* isoform. Crucially, because no *FoxP* null mutants have been generated, the relation between FoxP function and observed behavioral deficits still requires further characterization. Moreover, little is known about how FoxP may affect neuron morphogenesis and social behaviors. In this study, we generated null mutants, inducible overexpression lines, and GFP-tagged lines to characterize the impact of *Drosophila* FoxP in neuronal morphogenesis. In addition, we examined the importance of FoxP in cognition and social behaviors. Our findings allowed us to establish several parallels in FoxP function in the CNS between distantly related vertebrates and invertebrates.

## Results

### Conserved properties of mammalian FoxP proteins in *Drosophila*

The *FoxP* gene (CG43067, FBgn0262477) is the sole ortholog representing the human *FOXP* subfamily in *Drosophila melanogaster* (Fig 1A). To confirm *FoxP* isoform coding sequences we performed RT-PCR with the primer pairs 1–2 and 1–3, depicted in Fig 1B. Primer pair 1–3 amplified *FoxP-I2* and primer pair 1–2 amplified *FoxP-I1*, as well as high levels of *FoxP-I3* (Figs 1C and S1). The identities of all three isoforms were confirmed by Sanger sequencing.

To determine the dimerization properties of Drosophila FoxP, we performed a Y2H assay using the Drosophila FoxP N-terminus (aa 1–327) as a bait, which showed no self-activation. This bait, common to all three FoxP isoforms, isolated a FoxP fragment containing the leucine zipper domain (aa 194–338, prey). The construct was retransformed and retested using a β-galactosidase (β-gal) reporter assay, confirming the self-interaction (Fig 1D). Together, this shows that in Drosophila, similar to humans, different FoxP proteins are expressed and have dimerization capabilities.

### *FoxP* expression is highly enriched in the central nervous system and peaks after eclosion

**Fig 2:**
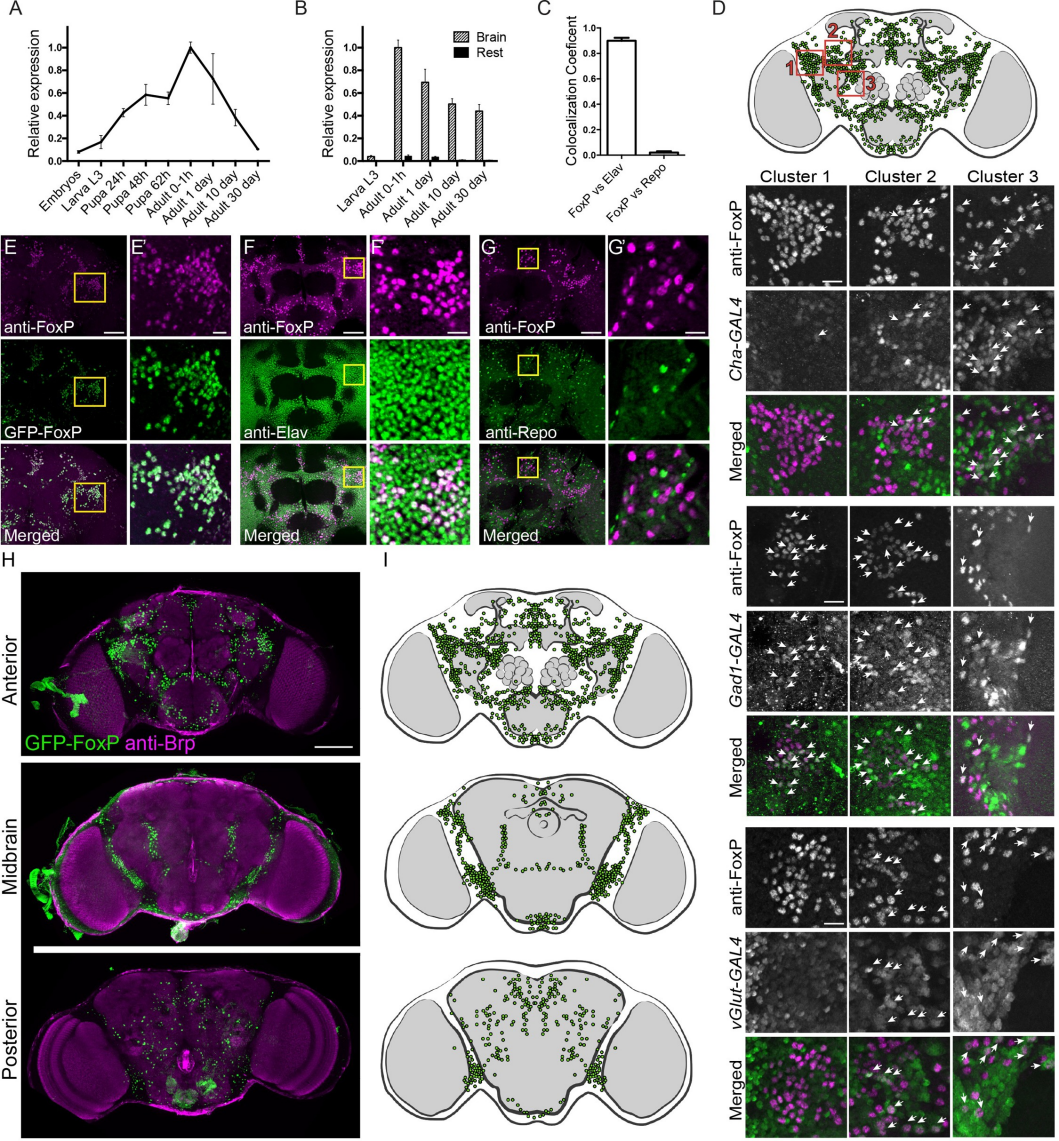
*FoxP* is expressed in approximately 1000 neurons of the *Drosophila* adult brain. (A) Graph represents relative *FoxP* expression levels over several developmental stages in wildtype flies. (B) Bars represent average relative *FoxP* expression in neural tissues (striped bars) and non-neural tissues (black bars) over different developmental stages. Data are represented as average and SEM of at least 3 biological replicates per developmental stage. (A, B) For the underlying numerical data see S2 Table. (C) Percentage of co-localization between anti-FoxP and anti-Elav signal or anti-Repo signal in wildtype male brains at 0–2 hours post-eclosion. Bars represent averages with SEM of a minimum of 4 brains. For the underlying numerical data see S3 Table. (D) Frontal brain schematic illustration of FoxP expressing neurons (green). The positions of clusters 1, 2 and 3 investigated for co-localization are highlighted by red squares. Clusters 1, 2 and 3 neurons of *w; Cha-*GAL4, *UAS-GFP*, *w; Gad1-*GAL4*/UAS-GFPnls and Vglut-*GAL4, w; *UAS-GFPnls/+ flies* co-immunostained with anti-FoxP (magenta) and anti-GFP (green). While maximum projections are shown, co-localisation was assessed on single optical sections. Arrows indicate co-localization. Scale bar corresponds to 10 μm (E-G) Maximum projection of *Drosophila* frontal brain image stacks. Scale bar corresponds to 50 μm. (E) *w;;GFP-FoxP* flies co-immunostained with anti-FoxP (magenta) and anti-GFP (green). (F) Wildtype flies co-immunostained with anti-FoxP (magenta) and anti-Elav (green) labeling neurons and (G) anti-Repo (green) labeling glial cells. (E’-G’) Magnification of E, F and G highlighted with a yellow square in the original images. Scale bar corresponds to 10 μm. (H) Maximum projection of image stack over a range of different brain depths showing the distribution of FoxP expressing neurons (green) together with the anatomical marker anti-nc82 (magenta) to visualize the different neuropils of *w;;GFP-FoxP* flies. Scale bar corresponds to 100 μm. (I) Schematic illustration of *FoxP*-expressing neurons (green) over the indicated brain sections. (Image stack is provided as S1 Video). Arrowheads indicate co-localization. Images were obtained from male brains at 0–2 hours post-eclosion.

We performed qRT-PCR to determine relative *FoxP* expression levels at various stages of *Drosophila* development (Fig 2A). *FoxP* expression progressively increased during development, presenting a 12-fold difference between its lowest levels, at embryonic stages, and its highest peak immediately after eclosion. During adulthood, *FoxP* expression progressively decreased. In thirty days old adults, *FoxP* expression levels were similar to those at embryonic stages (Fig 2A). To examine *FoxP* expression specifically in the CNS, we dissected and separated brains and thoracic-abdominal ganglia from the rest of the body tissues at different developmental stages and determined mRNA levels by qRT-PCR. *FoxP* was highly expressed in neural tissues compared to the rest of the fly body at all tested stages (Fig 2B). In L3 larvae, *FoxP* expression in neural tissues was 17-fold higher compared to non-neural tissues; this difference progressively increased with age, with a 54-fold difference between neural and non-neural tissues in thirty days old adults (S2 Table). These findings suggest predominant roles for *FoxP* in neural tissues.

We went on to characterize FoxP protein expression in the fly CNS by use of an anti-FoxP antibody previously generated by Lawton et al. [38] and a GFP-tagged FoxP line *w;;GFP-FoxP* (GFP-FoxP), generated in-house, which expresses GFP-tagged FoxP under the control of its endogenous regulatory elements. GFP-FoxP and anti-FoxP signal perfectly overlapped in *w;;GFP-FoxP* brains, validating both tools (Fig 2E). Both showed co-localization of FoxP with the neuronal marker anti-Elav (Figs 2F and S1), but not with the glial marker anti-Repo (Figs 2G and S1) in adult brains. We quantified the overlapping signal in *Drosophila* brain image stacks between anti-FoxP and anti-Elav, which was up to 90%, whereas only 2% overlap in signal was detected with anti-Repo (Fig 2C). These data indicate that FoxP is expressed in neurons but not in glial cells, agreeing with the conclusions of Lawton and collaborators [38].

GFP-FoxP expression and anti-FoxP immunostaining showed that FoxP is widely expressed in L3 larval brains, thoracic-abdominal ganglia and adult brains (Figs 2H and S2). Whole brain reconstruction and analysis of the total number of cell bodies indicated that FoxP is expressed in approximately 1000 brain neurons in young flies (one to two hours post eclosion). The anterior brain presented the majority of *FoxP* expressing neurons, organized in several clusters. In the midbrain, *FoxP* expressing neurons were mainly clustered in the ventrolateral neuropils. The posterior brain showed only scattered FoxP*-*expressing neurons (Fig 2H and 2I).

We next aimed to address in which neuronal subtypes FoxP is expressed. *UAS*-GFP was driven by GAL4 constructs specifically expressed in neuronal subsets representing the major neurotransmitters in *Drosophila* brain, and co-localization of anti-FoxP staining and GFP expression was studied. Since we found the FoxP expression pattern to be complex throughout the brain, we focused our attention on three clusters of FoxP-expressing neurons located in the frontal brain, as illustrated in Fig 2D. The GAL4 promoter lines *Cha-*GAL4 and *Gad1-*GAL4 were used to label cholinergic and GABAergic neurons, respectively, which represent the major excitatory and inhibitory neurons of the fly brain. Cluster three was mainly composed of cholinergic neurons, as revealed by a high degree of co-localization between anti-FoxP and *Cha-*GAL4*-*driven *UAS-GFP* labeling, whereas little co-localization was observed in clusters one and two. GABAergic neurons were abundant in clusters one and two, but not in cluster three. *Vglut-*GAL4 was used to visualize glutamatergic neurons. FoxP was present in several neurons labeled with *Vglut*-GAL4 in clusters two and three, but not in cluster one (Fig 2D). Neurons with less abundant neurotransmitters were visualized by UAS-GFP expression under the control of *Tdc2-*GAL4 (octopaminergic neurons), *Ddc-*GAL4 (dopaminergic and serotonergic neurons), and anti-TH staining (only dopaminergic neurons). Co-localization with FoxP staining was analyzed throughout the whole brain. No co-localization of FoxP and *Tdc2-*GAL4 was observed, indicating that FoxP is not expressed in octopaminergic neurons. Anti-FoxP and *Ddc-*GAL4 GFP signals co-localized in a single neuron, which upon immunostaining with anti-TH, was identified as dopaminergic (S3 Fig).

In conclusion, FoxP is present in cholinergic, excitatory and GABAergic, inhibitory neurons. Glutamatergic and dopaminergic neurons also express FoxP, although the latter was only observed in a single neuron. FoxP is present in scattered neurons but also in several clusters. In the latter, closely grouped *FoxP*-positive neurons often express the same neurotransmitter.

### Generation of FoxP mutants and transgenic lines

**Fig 3:**
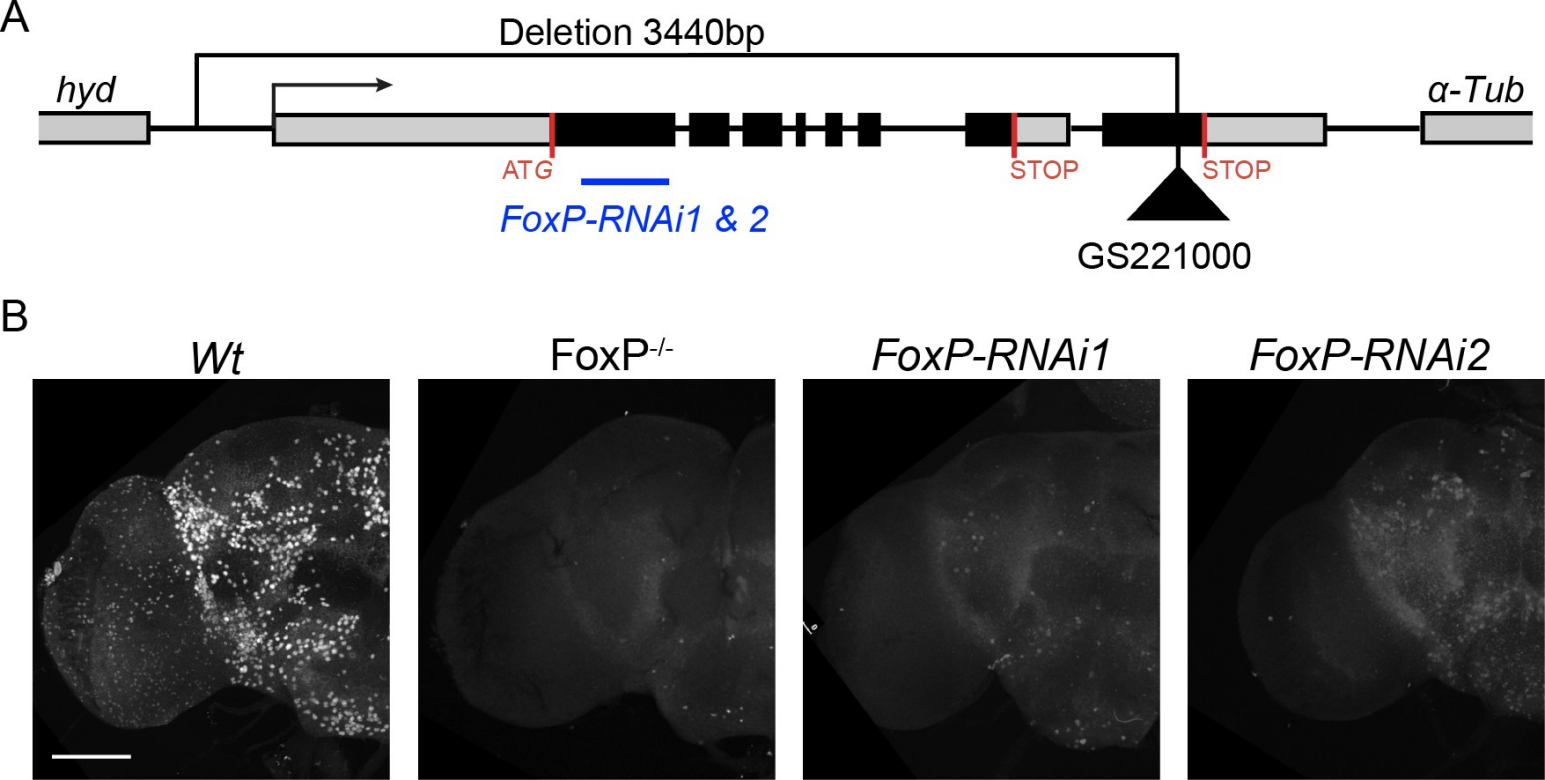
Characterization of *FoxP* mutants and RNAi lines. (A) Schematic representation of the *FoxP* genomic region and extension of the *FoxP* deletion. The transcriptional start site is indicated with an arrow, the two alternative stop sites with red lines. The original location of the P-element insertion *GS22100* is depicted with a black triangle. The *FoxP* sequence targeted by *RNAi1* and *RNAi2* is depicted as a blue line. Genes flanking *FoxP* on each side are also indicated. All three genes are oriented in the same direction. (B) Adult brain hemisphere of wildtype (Wt), *FoxP* null (*FoxP*^*-/-*^*)*, *w*, *UAS-Dcr2/Y; Actin-*GAL4*/+; UAS-FoxP-RNAi1* (*FoxP-RNAi1*)/+ and *w*, *UAS-Dcr2/Y; Actin-*GAL4*/+; UAS-FoxP-RNAi2/+* (*FoxP-RNAi2*) stained with anti-FoxP antibody. Scale bar corresponds to 50 μm. Images were obtained from male brains at 0–2 hours post-eclosion.

To provide further evidence for the specificity of the FoxP expression pattern and to enable characterization of FoxP functions, we generated *Drosophila* mutants (*FoxP*^*-/-*^) by transposon excision mutagenesis. Upon mobilization of the *GS22100* P-element located in exon 8 of the *FoxP* coding region, we isolated an imprecise excision event that deleted 3440 bp upstream of the original p-element insertion. (The sequence map can be found in S1 Text). The deletion removed nearly the entire *FoxP* gene, including the promoter region, exons 1–7 and most of exon 8, but did not affect the coding sequence of the flanking genes *α-Tub* and *hyd* (Fig 3A). The *FoxP* deletion allele was subsequently isogenized for 10 generations into the BestGene’s wildtype genetic background (see methods). This strain served as an isogenic control in all subsequent experiments, from now on referred to as wildtype (Wt). The deletion allele was balanced using a third chromosome balancer against the same genetic background and was found to produce few adult survivors, meaning that the deletion is mostly lethal.

Anti-FoxP immunohistochemistry was performed on adult brains of FoxP mutants. No signal was detected in *FoxP*^-/-^ adult brains (except a few foci of signal in the ventral brain which we conclude are due to antibody cross-reactivity), whereas staining recapitulated the above reported expression pattern in the wildtype (Fig 3B), validating both the expression pattern observed with the anti-FoxP antibody and the efficacy of the *FoxP* deletion allele. We also examined the presence of FoxP protein upon downregulation of *FoxP* expression with two inducible RNAi lines (*FoxP-RNAi1* and *FoxP-RNAi2*, targeting a region common to all three *FoxP* isoforms, Fig 3A), to probe the efficiency of these tools. Upon crossing them to the ubiquitously expressed *w*, *UAS-Dcr2; Actin-*GAL4 driver line, and carrying out brain dissections and immunostainings, we observed only residual signal in *FoxP-RNAi1 brains*. Compared to controls, *FoxP-RNAi2* brains consistently presented a stronger yet still clearly reduced signal, suggesting different levels of intermediate *FoxP* expression (Fig 3B). We conclude that both RNAi lines are able to knockdown FoxP protein levels, with *FoxP-RNAi1* being more efficient than *FoxP-RNAi2*.

We next generated UAS-*FoxP* lines specific to each of the three isoforms in order to individually drive their overexpression. We successfully created *UAS-FoxP* lines that could drive expression of *FoxP-I1* and *-I2*. For *FoxP-I3* several stable transgenes were generated but, for unknown reasons, these were unable to induce increased level of the transcript. The functional relevance of this isoform was therefore not further investigated.

### FoxP mutants present reduced life span and deficits in locomotion abilities

**Fig 4:**
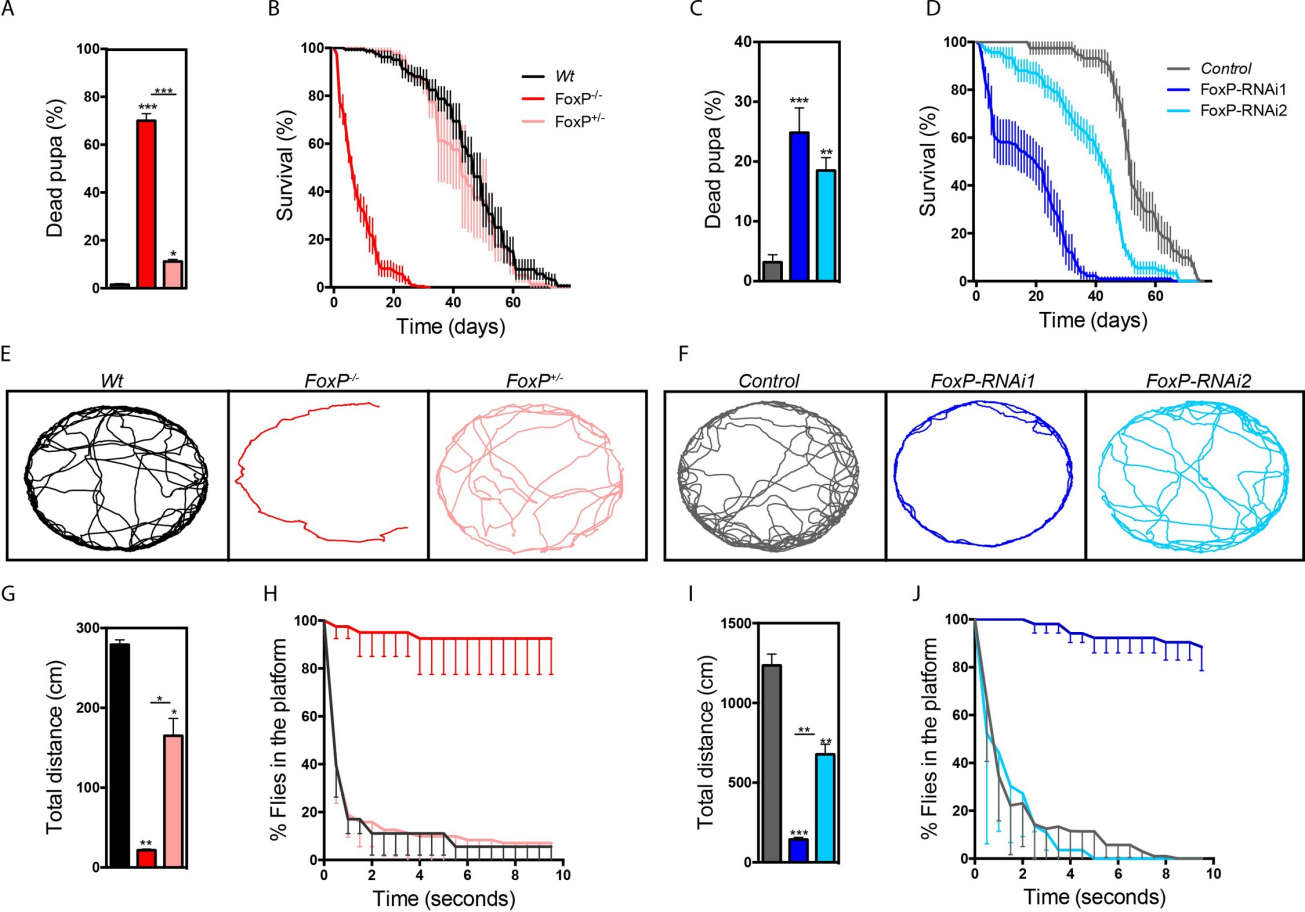
FoxP depletion leads to reduced fitness. (A, C) Fraction of dead pupa (in %). A minimum of 6 experimental replicates were analyzed per genotype. (B, D) Survival of males (in %) over days post-eclosion. A minimum of 4 experimental replicates were analyzed per genotype, with 15 male flies per experiment. (E, F) Locomotion trajectories of representative flies of the indicated genotypes. Male flies were recorded for 7 minutes at 10 frames per second in a circular arena (37 mm diameter). (G, I) Total distance (in cm) of walk in the 7 minutes of locomotion tracking. Data are represented as average and SEM of a minimum of 2 independent biological replicates per genotype. (H, J) *Drosophila* escape responses, assessed in the island assay. Graphs show % of flies that remain on the platform over time (10 seconds). Data are represented as average and SEM of 4 independent experimental replicates. The genotypes depicted in the graphs are: *FoxP* homozygous mutant (*FoxP*^*-/-*^, red), FoxP heterozygous mutant (*FoxP*^*+/-*^, pink), wildtype (*Wt*, black), *w*, *UAS-Dcr2/Y; Actin-*GAL4*/+* (Controls, grey), *w*, *UAS-Dcr2/Y; Actin-*GAL4*/+; UAS-FoxP-RNAi1/+* (*FoxP-RNAi1*, dark blue) and *w*, *UAS-Dcr2/Y; Actin-*GAL4*/+; UAS-FoxP-RNAi2/+* (*FoxP-RNAi2*, light blue). One-way ANOVAs with Tukey’s multiple comparison test were used to compare each condition and determine significant differences (*p<0.05, **p<0.01 and ***p<0.001). For the underlying numerical data see S4–S7 Tables.

To characterize FoxP function in *Drosophila* we first assessed the overall organismal fitness of homozygous and heterozygous FoxP mutants *(FoxP*^*-/-*^ *and FoxP*^*-/+*^, respectively). While no lethality was evident before pupal stages, the number of viable adult *FoxP*^*-/-*^ flies was dramatically reduced. Adult escapers only eclosed occasionally, and these were weak and sterile. The percentage of lethality at pupal stages was 70% for *FoxP*^*-/-*^ and 11% for *FoxP*^*+/-*^ animals, which was for both conditions significantly higher than in the wildtype flies (Fig 4A). Adult lifespan of eclosed *FoxP*^*-/-*^ flies was also dramatically reduced compared with wildtype flies. No differences in lifespan were observed for *FoxP*^*+/-*^ (Fig 4B). Similar results were obtained upon ubiquitous *FoxP* knockdown with the *Actin-*GAL4 driver line when crossed with either of the two *FoxP-RNAi* lines. Pupal lethality was also significantly increased for both RNAi lines and the average life span was decreased (Fig 4C and 4D). To measure locomotor abilities, we tracked spontaneous locomotion and flight escape responses. Spontaneous locomotion was tracked in an arena for 10 minutes. Both *FoxP*^*-/-*^ and *FoxP*^*+/-*^ flies walked significantly less distance compared to the controls (Fig 4E and 4G). Similar results were obtained with both *FoxP-RNAi* lines combined with an *Actin-*GAL4 driver (Fig 4F and 4I). Flight escape responses were assessed in the island assay [43]. These assay measures flight abilities by throwing flies onto a platform and determining the capacity to escape over time, healthy flies will escape the platform within few seconds whereas locomotor impaired flies might take longer or even be unable to leave the platform flying. *FoxP*^*-/-*^ and ubiquitous knockdown (*FoxP-RNAi1*) flies failed to escape from the platform, indicating a strongly reduced ability to fly. No significant differences were observed for *FoxP*^*+/-*^ and flies with ubiquitous knockdown induced with *FoxP-RNAi2* (Fig 4H and 4J). Finally, to determine whether the locomotor deficits observed in FoxP mutant conditions are of neuronal origin, as suggested by the predominantly neuronal expression pattern, we also downregulated FoxP with the panneuronal *elav*-GAL4 driver. Neuronal *FoxP-RNAi1* flies walked significantly less distance compared to the controls when spontaneous locomotion was tracked (S4A and S4B Fig). Similarly, a significant decrease in the flight response was observed upon neuronal knockdown using either of the RNAi lines in the island assay (S4C Fig). Together, these findings indicated that the locomotor defects are at least partly of neuronal origin. We conclude that FoxP is important for pupal and adult survival and locomotor abilities, in a dosage-dependent manner.

### FoxP mutants display morphological abnormalities of the mushroom body α-lobes

**Fig 5:**
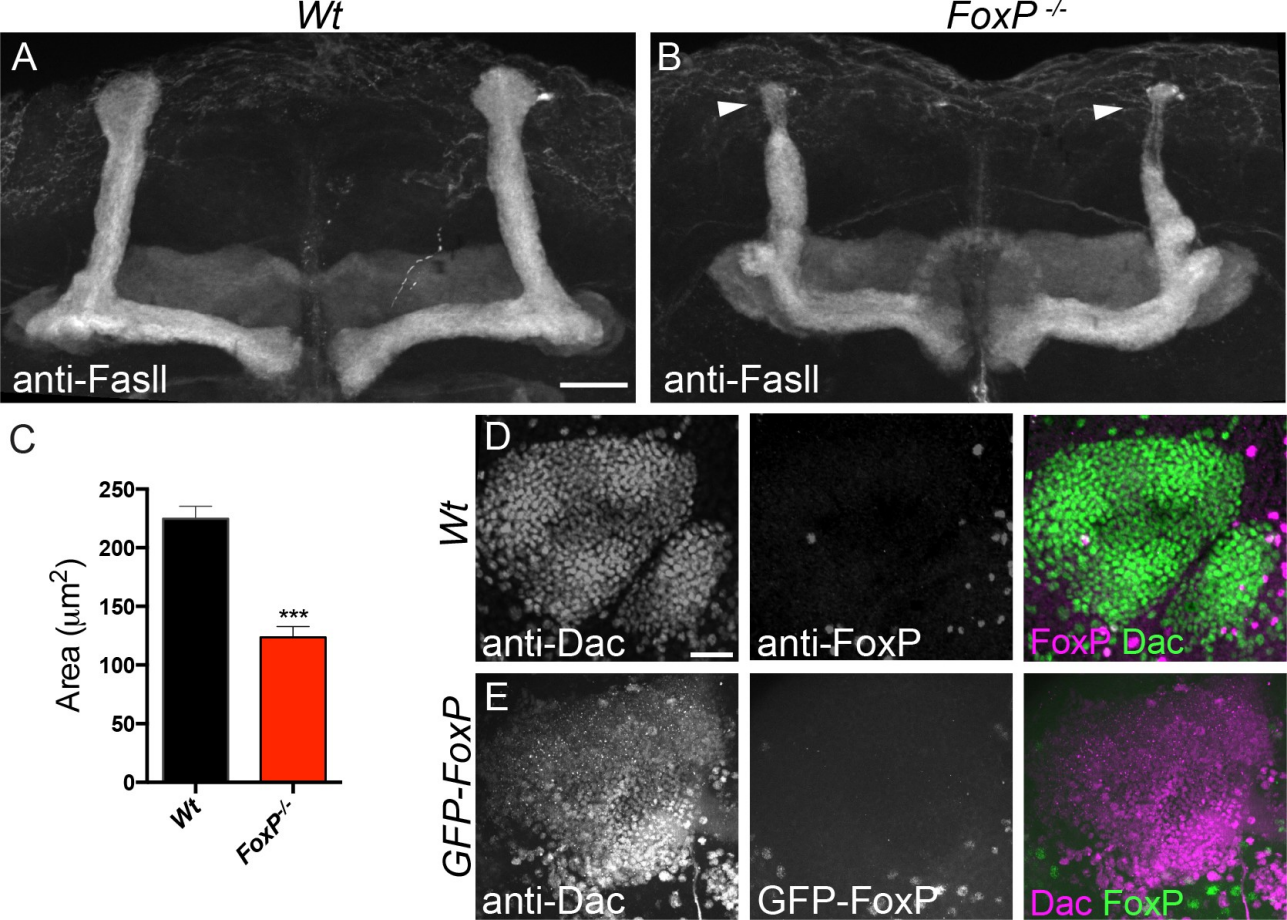
MB α-lobe morphology is affected in *FoxP*^-/-^ flies. **(A-B)** Maximum projection of MB image stacks of fly brains stained with anti-Fasll. Scale bar corresponds to 20 μm. (A) Wildtype and (B) FoxP mutants (*FoxP*^*-/-*^), arrowheads indicate MB α-lobes. (C) MB α-lobes area. Maximum projection of MB Kenyon cells, (D) Wildtype flies co-immunostained with anti-FoxP (magenta) and anti-Dac (green), (E) *w;;GFP-FoxP* co-immunostained with anti-GFP (green) and anti-Dac (magenta). Scale bar corresponds to 20 µm. Data are represented as average and SEM of a minimum of 31 α-lobes. T-test between conditions was performed to determine significance (***, p<0.001). Images were obtained from male brains at 0–2 hours post-eclosion. For the underlying numerical data see S8 Table.

FOXPs play key roles in development and function of neurons in higher organisms [8]. To determine if FoxP plays similar roles in the nervous system of *Drosophila melanogaster*, we first examined the overall brain morphology of *FoxP*^*-/-*^ mutants with the synaptic marker anti-Brp, but did not observe gross morphological abnormalities, although subtle volumetric changes were reported by Mendoza et. al. in FoxP^5-SZ-3955^ homozygous mutants, a P-element insertion which seems to affect only FoxP-I2 [42]. Immunohistochemistry with anti-Fasciclin ll (anti-Fasll) to visualize mushroom body (MB) architecture revealed a significant decrease in the area occupied by the thickened tips of MB α-lobes in the *FoxP*^*-/-*^ mutants compared to wildtype flies (Fig 5A–5C). This finding was interesting since putative expression of FoxP in the MB has previously been linked to perceptual decision-making [39, 40]. To determine whether FoxP is indeed expressed in MBs, we analyzed co-localization of FoxP labeling with anti-dachshund (Dac), a transcription factor expressed in the nuclei of Kenyon cells forming the MB. We did not observe any co-localization of either anti-FoxP or GFP-FoxP with anti-Dac, showing that FoxP is not expressed in Kenyon cells composing the MB in freshly eclosed adults (Fig 5D and 5E).

The morphological defect of MB α-lobes was recapitulated in *FoxP* panneuronal knockdown induced with *elav*-GAL4 driving either of the two *FoxP* RNAi lines (S5A–S5C and S5J Fig). The defect was also recapitulated by *FoxP* knockdown induced by the *247*-GAL4 MB driver with *FoxP-RNAi1*. *FoxP-RNAi2* showed the same tendency (S5D–S5F and S5K Fig). Finally, we overexpressed *FoxP* in the MB and observed a significant increase in α-lobe size (S5G–S5I and S5L Fig). These results indicate that FoxP expression levels determine the MB α-lobe size, likely in a cell-autonomous manner, indicating that FoxP is possibly expressed in MB Kenyon cells at developmental stages other than those investigated here.

### FoxP regulates neuromuscular junction postsynaptic morphology

**Fig 6:**
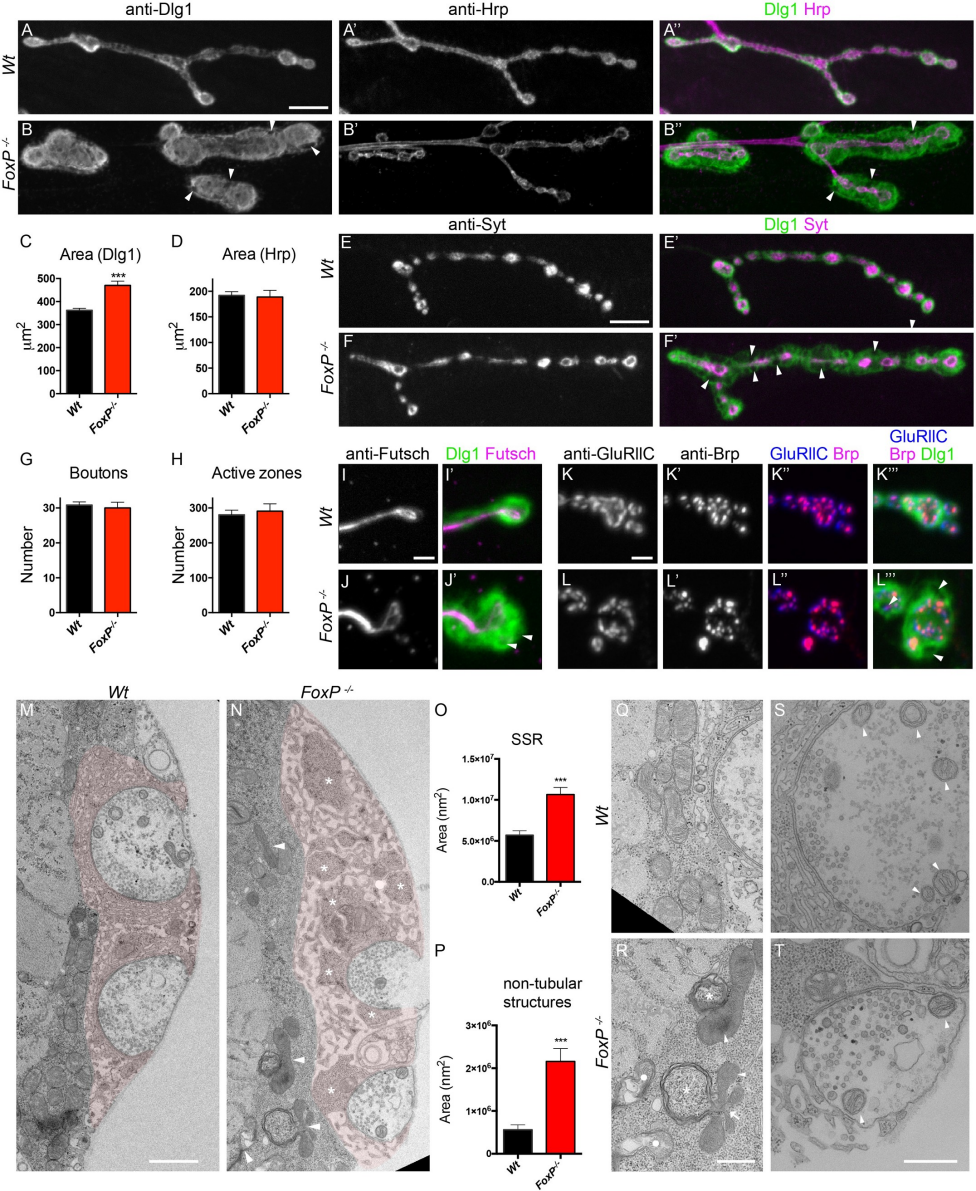
FoxP regulates NMJ postsynaptic morphology. Muscle four type 1b NMJs of *FoxP*^*-/-*^ mutant and wildtype wandering L3 male larvae. (A-B) Co-immunostaining of Dlg1 and Hrp. Scale bar: 10μm. Dlg1 staining showing a honeycomb-like pattern, disorganization and covering a wider region at *FoxP*^*-/-*^ mutant synaptic terminals compared to wildtype (*Wt*) terminals. (C) Dlg1 synaptic area is significantly increased in *FoxP*^*-/-*^ mutants (*wt* n = 56, *FoxP*^*-/-*^ n = 60). (D) Hrp-labelled synaptic area does not differ between *FoxP*^*-/-*^ and wildtype (*wt* n = 27, *FoxP*^*-/-*^ n = 23). (E-F) Co-immunostainings of Syt and Dlg1. Scale bar: 10μm. (G) The number of synaptic boutons (*wt* n = 55, *FoxP*^*-/-*^ n = 35) and (H) the number of active zones (*wt* n = 18, *FoxP*^*-/-*^ n = 19) do not differ between *FoxP*^*-/-*^ and wildtype. (I, J) Co-immunostainings of Futsch and Dlg1. Scale bar: 2μm and (K, L) Co-immunostainings of Brp, GluRllC and Dlg1. Scale bar: 2μm. Syt, Futsch, Brp and GluRllC NMJ immunolabeling appear normal in *FoxP*^*-/-*^. Electron micrographs of third instar larvae NMJ synaptic boutons (muscle 6/7), (M) wildtype and (N) FoxP mutants. SSR surrounding synaptic boutons was shaded in pale red using Adobe illustrator. Asterisks indicate non-tubular structures located in the SSR, arrowheads indicate defective mitochondria. Scale bar: 1 μm. (O) SSR area is significantly increased in *FoxP*^*-/-*^ (*wt* n = 39, *FoxP*^*-/-*^ n = 30). (P) The area occupied by non-tubular structures is significantly increased in *FoxP*^*-/-*^ (*wt* n = 40, *FoxP*^*-/-*^ n = 30). (Q-R) Mitochondria surrounding the SSR present ultrastructural defects in *FoxP*^*-/-*^ mutants. Arrowheads indicate defective cristae; arrows indicate multilobar mitochondria; asterisks indicate membranes folds around the mitochondria resembling autophagosomal structures, circles indicate collapsed mitochondria. Scale bar: 250 nm. (S-T) The conformation of neuronal mitochondria is unaffected in *the FoxP*^*-/-*^. Arrowheads indicate mitochondria. Scale bar: 500 nm. Bars represent the mean, error bars indicate SEM, t-test between conditions was performed for each parameter to determine significance (***, p<0.001). For the underlying numerical data see S9 and S10 Tables.

We went on to investigate potential roles of FoxP in synaptic development. We examined 3^rd^ instar larvae neuromuscular junctions (NMJ), a peripheral model synapse that allows detection of morphological anomalies at high resolution. Whereas presynaptic compartments, labeled by anti-horseradish peroxidase (Hrp), appeared normal in *FoxP*^*-/-*^ mutants, they presented striking defects in postsynaptic Dlg staining (Fig 6A–6D). In wildtype flies, the Dlg1 protein scaffold is distributed as a halo-like structure surrounding type I boutons in the postsynaptic region (Fig 6A). In *FoxP*^*-/-*^ mutants, the Dlg1 domain was strikingly larger compared to wildtype flies (Fig 6B and 6C). In addition, Dlg1 appeared in a disorganized, honeycomb-like pattern, and was absent from several focal areas within the enlarged postsynaptic compartment (Fig 6B, 6F’, 6K” and 6L”’ arrowheads).

In order to better understand the nature of the observed defects we first examined the NMJ with additional synaptic markers. NMJ bouton number and morphology were assessed upon immunolabeling with anti-Synaptotagmin (anti-Syt), a presynaptic vesicle associated protein. These analyses did not reveal significant differences between *FoxP*^*-/-*^ and wildtype flies (Fig 6E–6G). Presynaptic immunostaining of the NMJ microtubule scaffold with anti-Futsch appeared normal in *FoxP*^*-/-*^ mutants, including microtubule innervation of terminal boutons (Fig 6I and 6J), together showing that microtubule assembly and synaptic growth are normal in *FoxP*^*-/-*^ mutants. Further, the appearance and number of presynaptic release sites, so-called active zones revealed by anti-Brp labelling, were normal in *FoxP*^*-/-*^ flies compared to wildtypes (Fig 6H, 6K and 6L), as was proper apposition of active zone with postsynaptic glutamate receptor fields, revealed by anti-BRP and anti-GluRllC co-labelling (Fig 6K” and 6L”).

We further investigated the role of FoxP in synaptic architecture by electron microscopy. Consistent with the results from light microscopy we observed a significant increase in the area occupied by the subsynaptic reticulum (SSR) and the number of tubulolamellar infoldings of the muscle membrane surrounding synaptic buttons (Fig 6M and 6O). The SSR, typically formed by densely packed membrane folds, was less dense in FoxP mutants but contained numerous non-tubular structures (sarcoplasmatic patches not containing membrane infolds) (Fig 6N, asterisk), which occupied a significantly greater area in the FoxP mutants (Fig 6P). Surprisingly, while presynaptic mitochondria appeared normal, mitochondria surrounding the SSR of FoxP mutants, as well as mitochondria located elsewhere in the muscle, showed defective cristae and were severely affected in structure (Fig 6S and 6T). We observed several mitochondria that were fused and surrounded by layers of membranes, forming structures that could be autophagosomes (Fig 6Q and 6R).

To provide genetically independent evidence that loss of FoxP leads to an enlarged postsynaptic Dlg1 domain, we examined the effect of panneuronal knockdown using the two *FoxP* RNAi lines and the panneuronal *elav*-GAL4 driver. In both cases, we were able to replicate the significant increase in the area occupied by the Dlg1 postsynaptic domain (S6A–S6C and S6G Fig), but not the disturbed Dlg1 pattern observed in *FoxP*^*-/-*^ mutants. Upon downregulation of *FoxP* in muscles with the *Mef2-*Gal4 driver, we did observe the honeycomb-like pattern displayed by the *FoxP*^*-/-*^ mutants, which was more pronounced in *FoxP-RNAi1*. However, Mef2-driven knockdown did not significant increase the Dlg1 domain area (S6D–S6F and S6H Fig). Partial replication of the mutant phenotype with both panneuronal and muscle knockdown indicates that *FoxP* is required pre- and postsynaptically for normal NMJ growth and organization.

To determine whether increased levels of FoxP can alter synapse development, we also overexpressed *FoxP* isoforms using *elav*-GAL4 driver. This overexpression did not induce any significant differences in NMJ morphology (S12 Table). Our results indicate that FoxP is required for proper NMJ postsynaptic development, but increased expression levels cannot trigger NMJ changes.

### FoxP expression is required for dendritic morphogenesis in type lV dendritic arborization neurons

**Fig 7:**
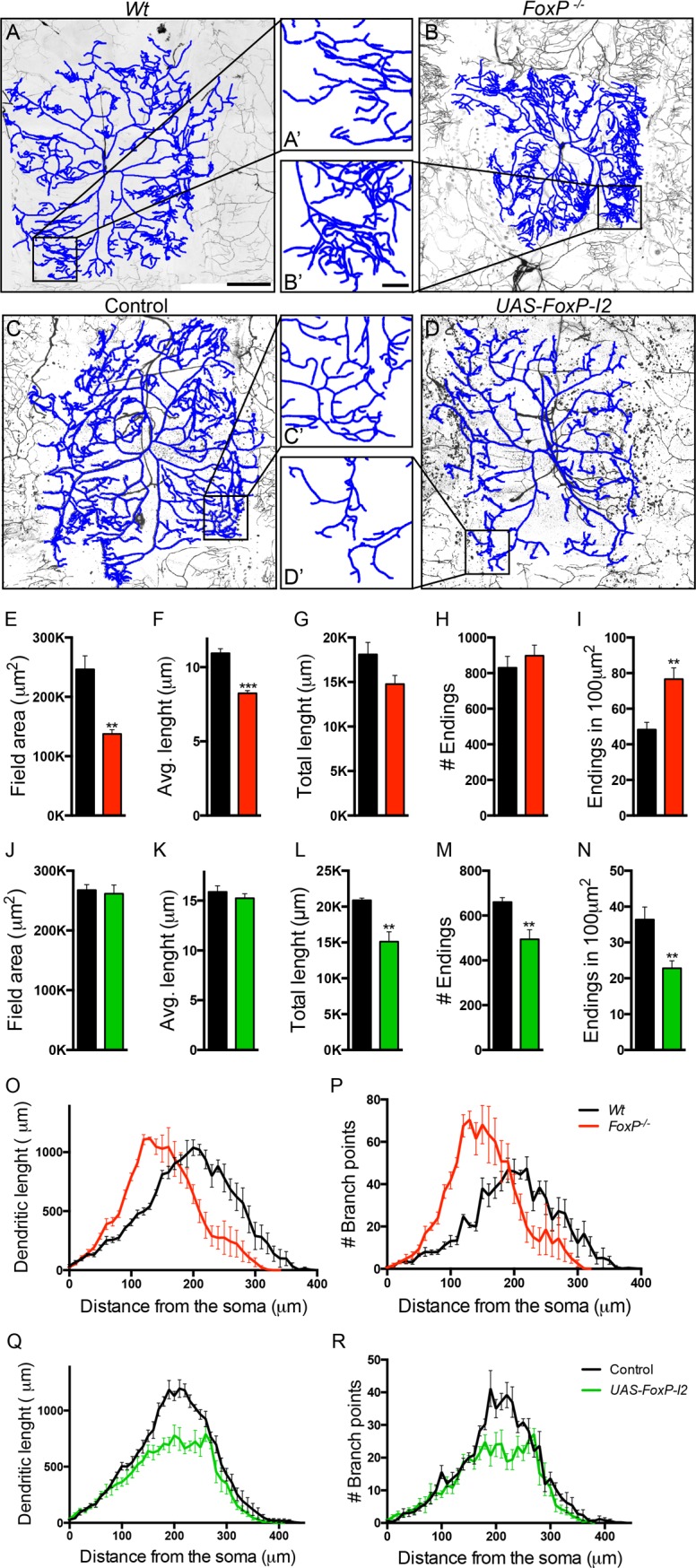
FoxP is required for dendritic growth and negatively regulates branching of type IV da neurons. (A-D) Confocal projections of class IV da neurons within segment A3 of third instar larvae, visualized with the class IV da-specific GFP expression (*477*-GAL4>*UAS-mCD8*::*GFP*). Reconstructions are represented in blue and are overlapping the original neuron. Scale bar: 100μm. (A’-D’) Magnification of dendrites as highlighted in the original image. Scale bar: 20μm. The following genotypes are depicted in the panels (A) *w w/Y; 477-*GAL4<*UAS-mCD8*::*GFP/+; +/+* (wildtype), *(B) w/Y; 477-*GAL4>*UAS-mCD8*::*GFP; FoxP*^*-*^ (*FoxP*^*-/-*^) (C) *w/Y; 477*-GAL4>*UAS-mCD8*::*GFP/+* (controls) and (D) *w/Y; 477*-GAL4>*UAS-mCD8*::*GFP/+; UAS-FoxP-I2/+ (UAS-FoxP-I2)*. (E-N) Quantitative analysis of dendritic trees, *FoxP*^*-/-*^ presents a decrease in (E) dendritic field area and (F) average branch length. (G) Cumulative branch length and (H) number of endings are unaffected. *Wt* (n = 5), *FoxP*^-/-^ (n = 5). (I) Dendritic endings density (number of endings in 100μm^2^) is increased in *FoxP*^*-/-*^. *Wt* (n = 9), *FoxP*^-/-^ (n = 9). *Wt* are depicted in black versus *FoxP*^*-/-*^ depicted in red. *UAS-FoxP-I2* presents unaffected (J) dendritic field area and (K) average branch length. (L) Cumulative branch length and (M) number of endings are decreased. Controls (n = 5), *UAS-FoxP*-*I2* (n = 5). (N) Dendritic endings density (number of endings in 100μm^2^) is decreased in *UAS-FoxP*-*I2*. Controls (n = 10, in grey), *UAS-FoxP*-*I2* (n = 10, in green). (O, Q) Sholl analysis of cumulative dendritic length; graph indicates the sum of dendritic length in concentric circles from the soma situated every 10μm. (O) *Wt* versus *FoxP*^*-/-*^ and (Q) control versus *UAS*-*FoxP-I2*. (P, R) Sholl analysis of cumulative number of branching points; graph indicates the sum of branching points located in concentric circles from the soma situated every 10μm. (P) *Wt* versus *FoxP*^*-/-*^ and (R) control (*w/Y; 477*-GAL4>*UAS-mCD8*::*GFP/+*) versus *UAS*-*FoxP-I2*. *Wt* (n = 5), *FoxP*^-/-^ (n = 5) controls (n = 5) and *UAS-FoxP*-*I2* (n = 5). Data are presented as averages with SEM. T-tests between conditions were performed for each parameter to determine significance (** p<0.01 and *** p<0.001). For the underlying numerical data see S13 and S14 Tables.

FOXP2 has been reported to regulate neurite outgrowth and normal maturation of dendrites of mouse cerebellar Purkinje cells [17, 18, 25]. To test for possible roles of *Drosophila* FoxP in dendritic morphogenesis, we examined the *Drosophila* class IV dendritic arborization (da) sensory neurons. Class IV da neurons present extensively branched dendritic arbors that cover the larval body wall [44]. Membrane-bound GFP was expressed in *FoxP*^*-/-*^ mutant class IV da neurons using the *477*-GAL4>*UAS-mCD8* Line. Dendritic trees of wandering third instar larva were imaged and manually traced to quantify several dendritic features. Loss of *FoxP* in *FoxP*^*-/-*^ larvae resulted in significantly smaller dendritic field areas and decreased average dendritic length (Fig 7A, 7B, 7E and 7F). The total dendritic length was slightly though not significantly decreased (p = 0.08) and the total number of dendritic endings was unaffected (Fig 7G and 7H). Sholl analyses (see methods) of *FoxP*^*-/-*^ class lV da neurons indicated an increase in dendritic length and branching points in the region more proximal to the soma and a decrease in dendritic length and branching in the more distal area (Fig 7O and 7P). All together the data indicate that the arbors of *FoxP*^*-/-*^ da neurons cover a smaller field but are more branched and compact within the covered area. This finding was confirmed by a significant increment in the number of dendritic endings in square areas of 100μm^2^ at the dendritic arbor periphery in *FoxP*^*-/-*^ (Fig 7I), also depicted in the magnification of the dendritic reconstruction (Fig 7A’ and 7B’). These mutant phenotypes were recapitulated by *FoxP* knockdown in type lV da neurons with *FoxP-RNAi1* (S7 Fig). These data validate the specificity of the phenotypes and suggests that they are due to loss of FoxP in class lV da neurons.

To further investigate the role of FoxP in dendritic morphogenesis, we overexpressed *FoxP* isoforms in the type lV da neurons. Whereas overexpression of *FoxP-I1* did not lead to any detectable phenotype (S8 Fig), overexpression of *FoxP-I2* significantly reduced the number of dendritic endings and total dendritic length (Fig 7C, 7D, 7J and 7K). The dendritic field area and average dendritic length remained unaffected (Fig 7L and 7M). Sholl analysis indicated a shorter dendritic length due to reduction in branch number over the dendritic tree (Fig 7Q and 7R). No differences were found in dendritic end radius between *FoxP-I2* and controls, suggesting that the dendritic area was unaffected. These results indicate that the dendritic trees present an area coverage that is normal but that they are less dense, with fewer branching points. Consequently, when measuring the dendritic ending density in areas of 100μm^2^ at the dendritic arbor periphery, a significant decrease in the number of endings was observed (Fig 7N and 7C’ and 7D’), opposite to the phenotype observed in *FoxP*^*-/-*^ mutants and upon *FoxP* down regulation. We conclude that FoxP regulates growth and shapes the morphology of class IV da neurons.

### FoxP regulates habituation learning

**Fig 8:**
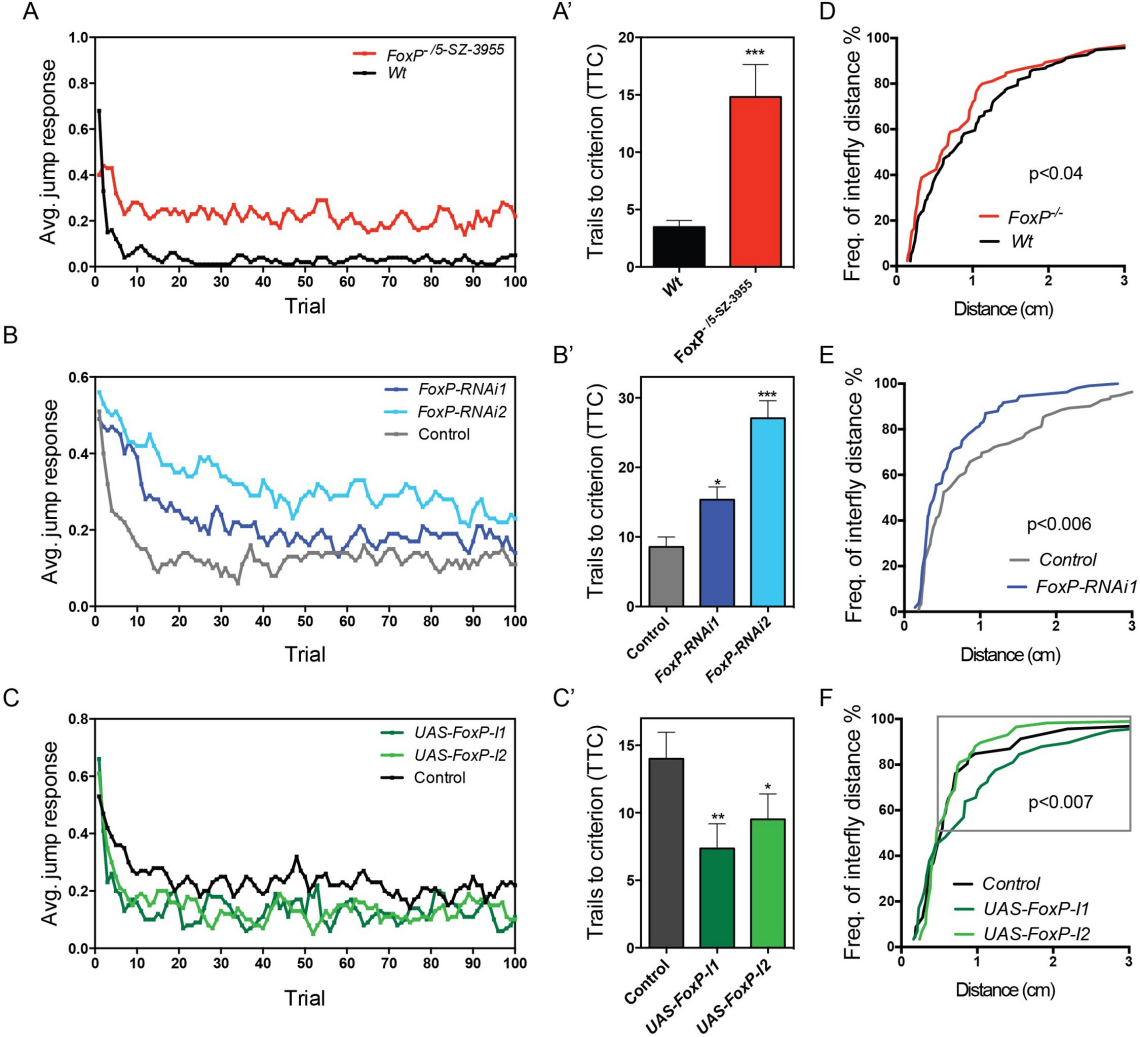
*FoxP* regulates light-off jump habituation and social distance. (A-C) Average jump responses (y axis) of three to six days old male flies from 3 independent experiments, with a minimum of 16 flies tested per experiment, plotted over 100 light off trails (x axis). (A) *FoxP*^*-/5-SZ-3955*^ mutant flies (*w; 2xGMR-wIR; FoxP*^*-/5-SZ-3955*^, in red) and wildtype flies (*w; 2xGMR-wIR; +/+*, in black) (B) *FoxP* panneuronal knockdown with *FoxP-RNAi1* (light blue) and *FoxP-RNAi2* (dark blue) (*w/Y; 2xGMR-wIR; elav-GAL4*, *UAS-Dcr2*/*FoxP-RNAi*) and control flies (*w/Y; 2xGMR-wIR; elav-GAL4*, *UAS-Dcr2/+*; in grey), (C) panneuronal overexpression of *FoxP-I1* (dark green) and *FoxP-I2* (light green) (*w/Y; 2xGMR-wIR/+; elav*-GAL4, UAS-Dcr2/ UAS-*FoxP*) and the respective controls (*w/Y; 2xGMR-wIR/+; elav*-GAL4, UAS-Dcr2/*+*; in black). (A’- C’) Mean number of trials to criterion (TTC) ± SEM of a minimum of 3 experimental replicates. T-tests or one-way ANOVAs with Dunn’s multiple comparisons were performed to assess differences between TTC of the different conditions (* p<0.05, ** p<0.01 and *** p<0.001). For the underlying numerical data see S17 Table. (D-F) Data of the social space assay are represented as cumulative relative frequency of the distance to the closest neighbor (Freq. of interfly distance). (D) *FoxP*^*-/-*^ mutants position themselves closer to each other than their Wt controls (Mann-Whitney, n = 136 *Wt* and n = 86 *FoxP*^*-/-*^ flies). (E) Panneuronal *FoxP* downregulation decreases social space (Mann-Whitney, n = 122 *w;UAS-Dcr2*/+; *elav-*GAL4*/+* and n = 108 *w;UAS-Dcr2*/+; *elav-*GAL4*/UAS-FoxP-RNAi1* flies). (F) Panneuronal overexpression of *FoxP-I1*, but not *Fox-I2*, increases social space at a distance >0.5 cm away from each other (grey rectangle). (Mann-Whitney, w;;*elav-*GAL4*/UAS-FoxP-I1* and w;;*elav-*GAL4*/UAS-FoxP-I2 n =* 58 flies each, and w;;*elav-*GAL4*/+* n = 45 flies).

Haploinsufficiency of FOXP1 leads to a neurodevelopmental disorder that among other features can include autism spectrum disorder (ASD) [45]. Haploinsufficiency of FOXP2 usually causes a speech disorder in absence of autistic features, and association of common variants with ASD is controversial [46, 47]. Nonetheless, several variants in Contactin-Associated Protein-Like 2 (CNTNAP2) gene, whose transcription is regulated by FOXP2 [48], are reported to confer an increased risk for ASD or ASD-related endophenotypes [49]. To determine whether *Drosophila FoxP* controls functions relevant for the associated human disorders, we investigated habituation with the light-off jump habituation paradigm. Habituation is a form of non-associative learning where an initial response to a repeated non-threatening stimulus decreases over time [50, 51]. It is an important prerequisite for higher cognitive functions and deficits have been repeatedly observed in individuals with ASD and in animal models of gene disruptions associated with ASD [52–54]. In the light-off jump habituation paradigm individual flies are exposed to 100 consecutive light-off pulses with a 1 second inter-pulse interval and their jump responses are measured. Habituation is quantified as the number of light-off pulses the flies need to reach no-jump criterion (TTC, see methods). Wildtype flies showed good initial jump responses to the light-off pulse (72% initial jumpers) and they quickly habituated to repeated pulses (Mean TTC = 15,06, Fig 8A), *FoxP*^*-/-*^ mutants were already unable to jump at the beginning of the assay (<10% initial jumpers), precluding the assessment of habituation. We used the *FoxP*^*5-SZ-3955*^ allele, containing the p-element *5-SZ-3955* inserted in the last exon of the *FoxP* coding region, to generate transheterozygous *FoxP*^*-/5-SZ-3955*^ animals. Using anti-FoxP immunostainings on brains of this genotype, we found this genetic condition to represent a hypomorph with residual *FoxP* expression similar to that observed when *FoxP* was downregulated with *FoxP-RNAi1* and the *UAS-Dcr2; elav*-GAL4 driver (S9 Fig). *FoxP*^*-/5-SZ-3955*^ flies qualified for assessment of habituation (80% initial jumpers). Compared to control flies with the same genetic background, they failed to efficiently suppress their jump response, maintaining a considerably higher jump response during the entire course of the assay (Fig 8A). Mean TTC of *FoxP*^*-/5-SZ-3955*^ flies was increased by 4-fold (Fig 8A’), revealing significant habituation deficits in *FoxP*^*-/5-SZ-3955*^.

We also subjected *FoxP-RNAi1* and *FoxP-RNAi2* panneuronal knockdown flies to the light-off habituation paradigm. Both RNAi conditions showed good initial jump response and recapitulated the habituation deficits observed in *FoxP*^*-/5-SZ-3955*^ (Fig 8B and 8B’).

We next overexpressed *FoxP* with *elav-*GAL4, to determine if this had an effect on habituation. Panneuronal *UAS-FoxP-I1* and *-I2* overexpression lead to a significant reduction in the mean TTC (Fig 8C and 8C’). Both conditions presented a slight decrease in their jumping abilities. We can therefore not exclude that the faster decline of the jump response is due to reduced fitness or fatigue.

In conclusion, the hypomorphic mutant and panneuronal *FoxP* knockdown flies exhibited deficits in habituation learning. These flies showed good initial jump responses to the light-off pulse, excluding sensory deficiencies, but they lacked neuronal plastic adaptation to suppress the jump reaction to repeated stimulation.

### FoxP regulates distance to the closest neighbor, a measure of social interaction

To determine whether *Drosophila* FoxP has an influence on social behaviors we performed the social distance assay. In this assay flies position themselves at a preferred distance to others in an undisturbed group of flies, and the average distance to the closest neighbor is determined [55]. This measure has been recently shown to be affected in *Drosophila* models with disruptions of ASD candidate genes [56, 57] and upon exposure to suspected environmental triggers of ASD [58]. The reported average distance that control flies establish with their neighbor is roughly around two-body lengths (0.5 cm for Canton-S) [55], which is also observed for our wildtype controls (Fig 8).

*FoxP* mutant flies presented a significant decrease in the average distance to the closest neighbor compared to their genetic control flies (Fig 8D). Similarly, significantly decreased average distance was also observed upon *FoxP* knockdown with panneuronal UAS-Dcr2; elav-GAL4 driver and *FoxP-RNAi1* (Fig 8E).

Finally, we overexpressed *UAS-FoxP-I1* and -*I2* using *elav-*GAL4 driver. No significant differences were found when considering the whole range of distances between *FoxP* overexpression flies and controls. However, when evaluating distances greater than 0.5 cm (50% of the flies), we found that flies overexpressing *FoxP-I1*, but not *FoxP-I2*, settled at further distances (Fig 8F).

## Discussion

In this study, we show that *Drosophila* FoxP is specifically expressed in about 1000 neurons in the adult brain and is required for many aspects of neural development, including synaptic morphogenesis at the NMJ, dendrite development of type IV multidendritic neurons and formation of MB α-lobes in the central brain. We also show that FoxP modulates normal locomotion and more complex behaviors such as habituation and social space. We report that *Drosophila* FoxP presents shared properties with its vertebrate orthologues including the conservation of protein domains and dimerization, and evolutionarily conserved functional properties in the CNS.

### FoxP, its potential expression and its function in the MBs

Previously, *Drosophila* FoxP has been proposed to contribute to perceptual decision-making, a behavior that involves MB αβ core (αβ_c_) Kenyon cells [39, 41]. It is conceivable that the size reduction in the MB α-lobes that we observed in FoxP mutants could underlie or contribute to deficits in this behavior. Despite this reduction in α-lobe size, we did not observe FoxP expression in MB Kenyon cells of young adults, neither with the anti-FoxP antibody nor with the GFP-tagged FoxP fosmid line. So far, three studies have investigated FoxP expression in the MB, with inconsistent results. Gupta and collaborators described FoxP expression in around 180 Kenyon cells innervating the αβ_c_ and γ-lobes [39]. Schatton and collaborators reported FoxP expression in Kenyon cells projecting to αβ_c_ lobes, but not in γ-lobes [40]. Finally, Lawton and collaborators found FoxP expression in neurons projecting to the protocerebral bridge, but not in the MB [38]. These prior studies used GFP expression driven by three independently generated *FoxP*-GAL4 promoter lines with promotor fragments of 1373 bp,1858 bp and 1532 bp upstream of the *FoxP* gene, respectively. The quality of such reporter-strategies depends on the organization of the *FoxP* promoter, which is not understood. Cis-regulatory elements located further away or downstream might be excluded. We attempted to circumvent this limitation by using a tagged fosmid line containing the *FoxP* genomic locus with its endogenous regulatory elements, and an anti-FoxP antibody that we validated on the *FoxP* mutant alleles. Both revealed a highly consistent expression pattern that did not overlap with MB Kenyon cells. This does not exclude the possibility that FoxP is expressed in αβ_c_ Kenyon cells at other developmental stages, for example at late pupal stage when αβ_c_ Kenyon cells are formed [59, 60]. MB expression at an earlier developmental stage is supported by the identified MB phenotype upon downregulating *FoxP* in MB Kenyon cells. The different *FoxP*-GAL4 promoter lines might retain the capacity to express FoxP in Kenyon cells, but could lack the elements to repress (or in other cases also promote) its expression at appropriate developmental stages. Further characterization of FoxP expression in αβ_c_ Kenyon cells across developmental stages could determine whether the morphological MB phenotype, which may either reflect axonal defects or a reduced amount of Kenyon cells, is caused by loss of FoxP in a MB-autonomous or non-autonomous manner.

### Regulation of dendrite morphogenesis by FoxP

During development, dendritic arbors change their morphology according to gene dosage and gene expression levels [61]. Interestingly, we observed different phenotypes upon depletion versus overexpression of FoxP in the type lV da sensory neurons of the *Drosophila* peripheral nervous system. These observations suggest that FoxP actively regulates dendrite morphological development and show that it needs to be accurately expressed to coordinate the appropriate dendritic developmental program. The FoxP transcriptional mechanism regulating dendrite development remains to be elucidated. FoxP may regulate expression of a single master regulator of dendrite morphogenesis that when overexpressed or downregulated leads to the appearance of different dendritic phenotypes. Alternatively, FoxP may regulate a transcriptional program comprising multiple target genes that regulate dendrite development; depending on the FoxP dosage, these genes and pathways may then lead to different phenotypes upon overexpression or down regulation.

### Regulation of synaptic organization by FoxP

Homozygous FoxP mutants displayed an enlarged and disorganized postsynaptic SSR at the NMJ. The SSR is composed by densely stacked muscular membrane sounding the type 1 NMJ boutons. The SSR also harbours the postsynaptic density (PSD), comprising a set of scaffolding proteins that recruit neurotransmitter receptors. FoxP might regulate NMJ postsynaptic formation through processes and pathways already described to regulate SSR growth and PSD formation. Several mutants have been described with phenotypes showing expanded and disorganized SSR resembling the FoxP phenotype. These include the scaffolding proteins Dlg-1 and its regulators that help to recruit diverse synaptic proteins and assemble them into large protein complexes [62–64], and membrane tubulation proteins, such as Syndapin or dRich, which induce or stabilize postsynaptic muscle membrane stacks to create the SSR [65, 66]. Signaling pathways, such as the wingless/wnt pathway [67], and other processes, such as ubiquitin homeostasis [68] or synaptic activity [69] are also critical for proper SSR growth. Often SSR overgrowth is accompanied by PSD anomalies such as defects in the distribution or composition of the glutamate receptor clusters[70], but we did not observe gross morphological abnormalities in the PSD after EM examination. We did observe severe defects in the mitochondria surrounding the SSR. Mitochondria in the muscles presented defective cristae, appeared fused and were surrounded by layers of membrane. An SSR overgrowth phenotype with mitochondrial defects has not previously been reported, but similar mitochondrial defects have been observed in cells that are undergoing apoptotic processes [71]. It is unclear whether the mitochondrial defects observed in the muscles are a direct consequence of FoxP loss or an early sign of organismal death. EM analysis was performed at late L3 larval stages and we documented 70% lethality in the FoxP homozygous mutants during metamorphosis (Fig 4A). Apoptotic processes might therefore already be present at larval stages, promoting the mitochondrial defects observed in EM. The finding that FoxP has lower expression levels in other tissues than in neurons may support a rather indirect effect on mitochondria in muscles. Nonetheless, it could be important to further investigate if FoxP regulates mitochondrial function in a direct and conserved manner.

In our study, we could not identify anomalies in the distribution of any of the presynaptic markers examined, indicating that the presynaptic site remains rather unaffected. Nonetheless, according to our results FoxP is required pre- and postsynaptically to ensure proper postsynaptic SSR growth. When FoxP was downregulated in neurons with the elav-GAL4 driver, an increase in the area occupied by the Dlg-1 compartment was observed, indicating that FoxP is required for trans-synaptic differentiation of the postsynapse [72, 73]. However, Dlg-1 distribution was disorganized upon postsynaptic downregulation of *FoxP* with the *Mef2*-GAL4 muscle driver correlating with the appearance of non-tubular structures in the SRR EM analysis, pointing to a potential postsynaptic role of FoxP. Finding that some aspects of the NMJ FoxP null mutant phenotype were recapitulated with the neuronal driver, whereas others were with the muscle driver suggests a complex origin of this phenotype. To dissect the underlying mechanisms will be an interesting topic for further studies. It should be noted that while the *Mef2*-GAL4 driver is highly expressed in muscles, its expression has also been reported in some parts of *Drosophila* CNS although (so far) it has not been detected in motoneurons [74].

### The functions of *Drosophila* FoxP from an evolutionarily point of view

In this study, multiple molecular properties of mammalian FOXPs were found to be conserved in *Drosophila*. First, the zinc finger, leucine zipper and forkhead DNA binding protein domains showed high homology between human and *Drosophila* FOXP*s*. We demonstrated that *Drosophila* FoxP can dimerize, which is crucial for mammalian FOXPs to bind DNA. In mammals, FOXP proteins either act as homo- or heterodimers with other FOXP isoforms and subfamily members, which modulates their specificity, leading to transcriptional control of different genes [34]. We observed that *Drosophila* FoxP, despite being represented by a single ancestral gene, can potentially be transcribed as three different isoforms that contain alternative forkhead DNA-binding domains. It is conceivable that therefore, similarly to the mammalian FOXPs, different dimers with differential transcriptional properties are formed in *Drosophila*. Some findings are in line with this theory. Overexpression of *FoxP-I2* in da neurons lead to a phenotype of reduced dendritic growth, whereas overexpression of *FoxP-I1* did not result in dendritic abnormalities. Conversely, overexpression of *FoxP-I1*, but not *Fox-I2*, led to increased social space. The *FoxP-I3* isoform lacks the forkhead DNA-binding domain, but contains the zing finger and leucine zipper domains required for dimerization. *FoxP-I3* may therefore be able to dimerize with the other two *FoxP* isoforms, whereby the lack of the forkhead domain may confer repressor properties. Truncated isoforms created by intron retention seem to be conserved throughout evolution, as described in other arthropods such as honey bees (*Apis mallifera*) [31], mice and even in humans [75]. In mammals, the existence FOXP2 isoforms that lack the forkhead domain have been reported and linked to mitochondrial functions [76], but some studies have proposed that these isoforms might be undergoing nonsense mediated-decay [75]. Unfortunately, we were not able to obtain expression of *FoxP-I3* from stable transgenic lines to study its function further.

There is some debate about the precise origin of the speech pathologies presented by human cases with heterozygous *FOXP2* mutations. During speech production, fine movements of the mouth and tongue have to be coordinated. The *FOXP2*-associated speech pathology might partially be caused by a defective regulation of fine motor skills, as supported by further problems of some (but not all) *FOXP2* mutation carriers in fine motor control (for example in buttoning clothes and tying shoelaces), although gross motor skills are normal [77]. Mouse *Foxp2* has been unambiguously linked with motor control [18, 21, 78]. Similarly, Lawton and collaborators proposed that *Drosophila* FoxP is important for behaviors requiring fine motor coordination, as downregulation of FoxP in neurons lead to sex-specific motor impairments in walking and flight [38]. We observed severe deficits in locomotor behaviors of *Drosophila* FoxP null mutants. In addition, heterozygous FoxP mutants showed milder defects in walking distance per time, correlating with the severity of the mutations. Similar correlations between mutations and the severity of locomotion phenotypes have been observed in Foxp2 mutant mice. Hemizygous/heteroyzgous knockout or mutation of Foxp2 causes fine deficits in motor skill learning and mild motor impairments whereas homozygous disruptions lead to severe motor impairments [18, 21, 25]. The motor deficits observed in *Drosophila* FoxP mutants could result from the observed mitochondrial anomalies in the muscle cells. Nonetheless, knockdown of FoxP in neurons is sufficient to cause motor impairments, indicating that these deficits are at least partially caused by a neuronal component. These results are in agreement with a recent mouse study in which conditional, brain-specific Foxp2 knockouts cause distinct types of motor-skill deficits [79].

*FOXP* genes are important regulators of development and are expressed in the developing brain [80–82]. In other arthropods, including honey bees, FoxP is abundantly expressed in the CNS but its expression is low during development and progressively increases with age [31, 83]. Our results show that *Drosophila* FoxP expression levels are low during embryogenesis, but increase dramatically during pupal development. During metamorphosis, the *Drosophila* brain undergoes important remodeling processes including neuronal pruning, axonogenesis, neurite outgrowth and synaptic formation [84, 85], processes already described to be modulated by FOXPs in other organisms [16–19]. We also observed defects in dendrite outgrowth in *Drosophila* FoxP mutants, indicating that FoxP is important for dendrite morphogenesis. Mouse Foxp2 and human FOXP2 are required for dendrite development of Purkinje cells, neurite outgrowth of striatal precursors and dendritic morphogenesis [16–19], indicating evolutionarily conserved contributions of FOXPs to these processes that extend to invertebrates.

*Drosophila* FoxP is predominantly expressed in the CNS, with minimal expression in other tissues (Fig 3B). By contrast, in mice the various *Foxp* genes are also expressed and play crucial roles in other organs such as the heart and lungs [18, 21, 86]. Whether *Drosophila* FoxP has functions in other tissues remains to be addressed. In the CNS, *Drosophila* FoxP is expressed in a specific subset of neurons, similarly to vertebrate FOXPs, which are expressed only in certain brain regions regions to modulate their development [26, 28, 87]. Equally, we also observed that *Drosophila* FoxP modulates the development of certain brain structures such as MB α-lobes.

Foxp1 and 2 are expressed in projection neurons of the cerebral cortex [88], which are excitatory glutamatergic neurons. Foxp1 and 2 are also expressed in, striatal medium spiny neurons [17, 89] and V1 interneurons of the spinal cord that use GABA and/or glycine as inhibitory neurotransmitters [90, 91]. Similarly, our findings reveal that *Drosophila* FoxP is expressed in neurons secreting several different neurotransmitters including those that secrete acetylcholine (the main excitatory neurotransmitter of the *Drosophila* brain), glutamate or GABA.

We observed defects in the NMJ postsynaptic compartment in *Drosophila FoxP* mutants, revealing a novel role for this gene. To our knowledge, this is the first study that demonstrates that FOXPs are involved in synapse formation. However, FOXPs are known to play roles in synaptic transmission and plasticity in mice [14, 19, 21]. Alterations in synaptic morphology can determine the functional aspects of a neuronal network and may underlie observed defects in synaptic transmission efficacy and plasticity. Synapse anomalies have been put forward as a potential central theme in neurological and psychological disorders such as autism spectrum disorders and intellectual disability [92]. Thus, specifying the contributions of FoxP to shaping of synapse morphology in *Drosophila* and other animal models may lead to a better understanding of pathologies associated with FOXP1/2 disruptions.

*Drosophila* FoxP is required for light-off jump reflex habituation, a simple form of non-associative learning. Habituation, a mechanism crucial for sensory filtering, is evolutionarily conserved across the animal kingdom. It is one of the simplest and earliest forms of behavioral plasticity manifesting after birth [93]), and is thought to provide the foundation for higher cognitive functions [94]. Several studies report that ID/ASD patients show defective habituation in neural activity or behavior [52, 95–97]. These defects might contribute to symptoms associated with defective cortical filtering, such as sensory hypersensitivity and other ASD features [98, 99]. In recent years, studies in *Drosophila* but also in other organisms have reported habituation deficits as a widely affected mechanism in models of ID/ASD disorders [100, 101]. Whether habituation deficits contribute to cognitive and learning defects observed in FOXP patients remains to be investigated. Furthermore, we observed a role for *Drosophila* FoxP in regulating social space, a feature also disrupted in ASD patients [102]. Other proteins that regulate social space in *Drosophila* are e.g. the synaptic protein Neurobeachin, an anchor protein that is homologous to the autism candidate gene *NBEA* [56] and the pre-synaptic proteins involved in dopaminergic synthesis and release [103, 104], homologues of the vesicular monoamine transporter (VMAT) and Tyrosine hydroxylase (TH) involved in dopamine synthesis. Both have been associated with ASD [105, 106] and other neurodevelopmental disorders [107–109]. Foxp1 brain-specific KO mice are also reported to show features associated with ASD such as repetitive behaviors [14].

Evidence that FOXP2 was subject to selection within the last ~100K years of human evolution [110] has recently been refuted by new genomic information [111], but the arguments for an important role of FOXP proteins in human brain development and function remain. Although the underlying mechanisms are still incompletely understood, our study demonstrates that this importance extends to invertebrates. We here established a series of *Drosophila* FoxP properties and phenotypes that make *Drosophila* an attractive model to further dissect the molecular basis of FoxP action in the healthy and diseased nervous system. Our study reports a spectrum of FoxP neuronal functions, from the molecular to the cellular and behavioral level. Several of these are novel while others parallel previous findings in other species, illustrating that FoxP neuronal function extends beyond vertebrates and is highly conserved.

## Methods

### Nomenclature

As proposed [112], references to human winged helix/forkhead transcription factors are written in capitals (FOXP2), references to mice proteins are starting with a capital letter followed by lower case letters (Foxp2) and references for all other organisms are symbolized as FoxP. Italics is employed when referring to gene symbols or mRNA. Roman type refers to proteins or both, genes and proteins.

### Fly stocks and maintenance

Flies were raised on standard medium (cornmeal, sugar, yeast). The following genetic strains where obtained from the Bloomington stocks center (Indiana University): *w;; elav-*GAL4 (8760)*; 477*-GAL4>*UAS-mCD8*::*GFP* (8746); *w; Actin-*GAL4 (4414); *w; Mef2*-GAL4 (27390); *w; 247*-GAL4 (50742); *y,w;; Ki P[53]99B* (4368); *w; Gad1-*GAL4 (51630)*; w; Cha-*GAL4, *UAS-GFP* (6793); *Vglut-*GAL4, *w* (24635); *w; Ddc-*GAL4 (7010)*; w*, *Tdc2-*GAL4 (9313) and w; *UAS-GFPnls* (4775). FoxP RNAi lines 15735 and 15732, referred as *FoxP-RNAi1* and *FoxP-RNAi2* respectively, the corresponding genetic background control (60000); *w*, *UAS-Dcr2* (60008) and *w; UAS-Dcr2* (60009) were obtained from the Vienna Drosophila Resource Centre (VDRC). P-element insertion lines *y*,*w; FoxP*^*GS22100*^/TM3,Sb,Ser (203643); y,w; *FoxP*^*5-SZ-3955*^ (126252) where obtained from the Kyoto Stock Center (DGRC). The in-house line *w; 2xGMR-wIR; elav-*GAL4, *UAS-Dcr2* (described in [113]) was used in habituation experiments. It contains two copies of an constitutively expressed eye-specific RNAi construct targeting the gene, to suppress the eye pigmentation as required for light-off jump response.

Fly stocks, crosses with *Actin-*GAL4 driver and all crosses for habituation were maintained at 25 degrees, 70% humidity. Other crosses using the UAS-GAL4 system to manipulate gene expression, were performed at 28 degrees, 60% humidity. All flies were raised in a 12:12 hours light dark cycle.

### Generation of a *FoxP* excision mutant, *UAS-FoxP* transgenes, and a GFP-tagged *FoxP* fosmid

*FoxP* mutants were obtained upon P-element mobilization in y,w; *FoxP*^*GS22100*^*/TM3*,*Sb*,*Ser*. Females were crossed to *y*,*w;; Ki*,*P{Δ2–3}99B* males to induce mobilization of the GS22100 P-element. In the F1 generation *y*,*w;; FoxP*^*GS22100*^*/Ki P{Δ2–3}99B* males were selected and crossed with y,w;; Sb/TM3 y+ Ser females. Male progeny with white eyes, indicating excision of GS22100 P-element, were isolated. Two hundred independent excision lines were screened by genomic PCR and sequenced to determine the excision sites (primers are indicated in S1 Table). Lines carrying a deletion in the *FoxP* genomic region were isogenized for 10 generations with the Bestgene wildtype genetic background, a background that has been derived by exchanging the w- allele of the strain that the company regularly uses to inject P-element-based transgenes by a w+ allele.

*FoxP* isoforms 1 and 2 were amplified from cDNA obtained from fly brains with the primers indicated in S1 Table (see mRNA extraction and cDNA synthesis section) and cloned into UASp vector from The *Drosophila* Gateway Vector Collection (https://emb.carnegiescience.edu/drosophila-gateway-vector-collection#Brand_A._H._and_Perrimon_N.). After sequence verification, transgenesis was performed by BestGene (https://www.thebestgene.com) according to standard procedures to create inducible *FoxP* isoform 1 and 2 overexpression lines (*UAS-FoxP-I1* and *UAS-FoxP-I2*).

A line expressing FoxP tagged with GFP was generated using the fosmid FlyFos022188 from the *Drosophila melanogaster* genome-wide fosmid library (https://transgeneome.mpi-cbg.de). The genomic element FlyFos022188 is expanded 24,319bp upstream and 2111bp downstream the *FoxP* gene. A 2XTY1-sGFP-V5-preTEV-BLRP-3XFLAG tag was introduced in the N-terminal FoxP region of the fosmid, immediately after the FoxP start codon with the primers indicated in S1 Table. Transgenes were generated using the host strain y[1], w[*], P{nos-phiC31int.NLS}X; PBac{y+-attP-3B}VK00033 (BL-32542) and previously established procedures [114, 115] to generate the transgenic line FlyFos022188(pRedFlp-Hgr)(CG16899[13132]::2XTY1-SGFP-V5-preTEV-BLRP-3XFLAG)dFRT. This line will be referred to as w;;*GFP-FoxP* throughout the manuscript.

### Pairwise protein sequence alignment

Percentage similarity of amino-acid sequences between FoxP protein domains (leucine zipper, zinc finger, and forkhead) of human FOXP1, 2 and 4 and *Drosophila FoxP* was calculated using optimal global alignment of pairwise sequences. The conservation scoring was performed by PRALINE (http://www.ibi.vu.nl/programs/pralinewww/).

### Yeast two-hybrid assay (Y2H)

The GAL4-based yeast two-hybrid system (HybriZAP, Stratagene, La Jolla, USA) was used for identifying protein interaction partners of FoxP. A DNA construct coding for FoxP amino acids 1–327 (common to all isoforms, excluding the forkhead domain) was fused to a GAL4 DNA-binding domain with Gateway technology and was used as bait. A normalized, universal *Drosophila* cDNA Mate & Plate library (Clontech Laboratories inc., Mountain View, USA) was used to perform the Y2H screen as previously described by Letteboer and Roepman [116].

### mRNA extraction and cDNA synthesis

Samples were collected and transferred to RNA*Iater* solution (Sigma). A total of ten adult or larval brains were dissected per sample. For other samples three larvae, pupae, adults, or their remainders after brain removal, were used. Total RNA was extracted using the Arcturus PicoPure RNA Purification kit (Thermo Fisher Scientific). To avoid amplification from genomic DNA, DNase treatment was performed using the DNA-free Ambion kit and RNA was reverse transcribed into cDNA using the iScript cDNA (Bio-Rad) according to manufacturers’ procedures.

### Quantitative real-time PCR (qRT-PCR)

Quantitative PCRs (qPCRs) were performed using the GoTaq qPCR Master Mix (Promega) on an ABI 7500 Fast Real-Time machine. The following cycling conditions were used: initial denaturation for 3 min at 95°C, followed by 15 s at 95°C and 30 s at 60°C for 40 cycles (QPCR data collection). The products were then denatured at 95°C for 1 min, cooled to 65°C for 1 min (melt curve data collection). The primers amplifying *FoxP* (common region for all isoforms) and the reference genes *RNApol2* and *1tub23cf* are provided in S1 Table. For each condition, three biological and two technical replicates were analyzed. Differential gene expression was calculated using the 2^ΔΔCt^ method [117]. The average Ct value for each sample was calculated and subtracted from the geometric mean Ct value of the reference genes *RNApol2* and *1tub23cf* in order to calculate the ΔCt value [118].

### PCR

Primers for amplification of FoxP isoforms were designed with Primer3 (http://bioinfo.ut.ee/primer3-0.4.0/). Amplification by PCR was performed on 40 ng of cDNA with Taq DNA polymerase Amplitaq (Life Technologies). Primer sequences are provided in S1 Table. PCR fragments were purified with NucleoFast 96 PCR plates (Clontech) in accordance with the manufacturer's protocol. Sequence analysis was performed with the ABI PRISM BigDye Terminator Cycle Sequencing V2.0 Ready Reaction kit and analyzed with an ABI PRISM 3730 DNA analyzer (Applied Biosystems).

### Brain immunohistochemistry

FoxP immunostainings were performed in wildtype animals or in the GFP-tagged *FoxP* animals. Third instar larvae or freshly eclosed adult males were collected. Brains were dissected and fixed in cold methanol for 2 minutes, washed with PBS-T (PBS (phosphate-buffered saline) containing 0.3% Triton X-100) for 1 hour and blocked with PBS-T 10% normal goat serum (NGS) for 2 hours. Brains were incubated with primary antibodies in PBS-T 10% NGS for 2 nighs at 4°C: guinea pig-anti-FoxP (1/300) (gift from D. Deitcher [38], rat-anti-Elav (1/100), mouse-anti-Repo (1/100), mouse anti-Dac (1/100), mouse-anti-Fasll (1/10), mouse-anti-nc82 (1/50) (Developmental Studies Hybridoma Bank, University of Iowa), rabbit-anti-GFP 1/600 (A11122, Invitrogen), rabbit-anti-TH 1/100 (Ab 152, Millipore). After primary antibody staining, brains were washed five times in PBS-T at room temperature (RT). Secondary antibodies were diluted 1/500 in PBS-T 3% NGS and incubated for 38h hours at 4°C: goat anti-rabbit Alexa Fluor 488 (A11008, Molecular Probes), goat anti-mouse Alexa Fluor 488 (A11029 Life technologies), goat anti-rat Alexa Fluor 488 (A11006, Molecular Probes), goat anti-guinea pig Alexa Fluor 568 (A11075, Invitrogen). Finally, brains were washed five times in PBS-T at RT and mounted with ProLong Gold Antifade Mountant (Thermo Fisher). Images were obtained with Zeiss AxioImager Z2 fluorescent microscope equipped with an ApoTome (Carl Zeiss B.V.).

### Co-localization

Co-localization between two channel image stacks was determined using JACoP (https://imagej.nih.gov/ij/plugins/track/jacop2.html) [119] plugin for FIJI [120] (https://fiji.sc). Manders' overlap coefficient was used to determine the degree of co-localization on single optical sections. A value of 0 indicates no overlap and a value of 1 indicates 100% co-localization of signals.

### Analysis of mushroom body morphology

Mushroom bodies were visualized with anti-Fasll. Area, width and length of mushroom body α-, β-, and γ-lobes were manually measured in FIJI (https://fiji.sc) [120] using the free hand selection tool. Statistical significance was analyzed using the One-Sample T-Test in GraphPad Prism (version 5.00, GraphPad Software).

### Survival experiments

Flies were transferred to fresh food every 3 days. Survival was monitored daily. A minimum of four vials with 10 to 15 flies were monitored per genotype.

### Locomotor assays

Tracking arenas were modified lids of 10 cm petri dishes, with a height of 4 mm to allow flies to freely walk but not to jump or fly. Male flies were collected the day of eclosion and transferred to a new 10ml vial containing standard food. Tracking was carried out the next day under constant conditions (25°C, 70% humidity). Experimental flies were transferred to the arena using an aspirator and were allowed to acclimatize for 5min. Next, 10 min videos (10 fps) were recorded using HandyAvi software (Azcendant) connected to a Logitech C525 webcam positioned above the center of the arena. Locomotion was tracked using the semi-automatic machine-vision program Ctrax (Version 0.5.6) [121]. Distances within the videos were calibrated based on a known measure. Ctrax output files were further analyzed in Excel to calculate total distance and average velocity. For each genotype, the experiment was repeated 3 times. Significance was calculated using a two-tailed t-test.

### Island assay

The island assay was conducted as previously described [43]. In brief, three to four days old male adult flies were thrown to a platform and their flight escape response was video recorded. The number of flies remaining on the platform was quantified every 0.5 seconds. GraphPad Prism was used for the statistical comparisons. All behavioral experiments were performed at room temperature under standard light conditions.

### NMJ immunohistochemistry

Third instar male larvae were collected and dissected in 1xPBS and fixed in 4% paraformaldehyde for 1h or in cold methanol for 2min. Larvae were washed twice shortly in 1xPBS-T, and incubated with the primary antibodies mouse-anti-Dlg1 1:100 (Developmental Studies Hybridoma Bank) (DSHB)), mouse-anti-nc82 1:40 (DSHB), mouse-anti-22c10 1:100 (DSHB), rabbit-anti-Hrp 1:500 (Jackson ImmunoResearch), rabbit-anti-Syt (kindly provided by H.Bellen) 1:2000 and mouse-anti-GluRllC (kindly provided by DiAntonio) in 1xPBS-T over night at 4°C. Secondary antibodies goat-anti-rabbit Alexa-568 1:500 (Molecular Probes) and goat-anti-mouse Alexa-488 1:500 (Life technologies) were diluted in PBS-T, and incubated for 2 hours at room temperature. For the triple staining nc82/GluRIIC/Dlg1, Dlg1 was visualized conjugating the primary antibody anti-Dlg1 (1:25) with the Zenon Alexa Fluor 647 Mouse IgG1 labeling kit (Invitrogen) according to the manufacturer’s protocol. Larvae were placed on microscope slides and mounted in ProLong Gold Antifade Reagent (Molecular probes). Images were obtained with the Zeiss Axio Imager 2 using the ApoTome. Obtained images were analysed with the macros *Drosophila*_NMJ_Bouton_Morphometrics and *Drosophila*_NMJ_ Morphometrics macro in FIJI [120, 122, 123]. GraphPad Prism software was used for statistical analysis.

### Electron microscopy

Open book preparations of third instar larvae were fixed in 4% PFA and 0.5% GA for 10 min, followed by 1 h fixation in 2% GA in 0.1 m sodium cacodylate buffer at room temperature, and a washing step in cacodylate buffer for one hour at room temperature. The larvae were postfixed on ice in the dark with 1% OsO_4_ in 0.8% KFeCN, washed with NaCac buffer for 1 h on ice and stained with 1% uranyl acetate (UAc) on ice. Subsequently, the preparations were dehydrated in ethanol, followed by ethanol/epon solution and finally in pure liquid Epon overnight. Then the samples were embedded in a single drop of Epon. Muscle 6/7 of abdominal segments 2 to 5 were dissected, and multiple muscles were stacked and embedded in an Epon block, which was heated overnight at 60°C for polymerization. Ultrathin sections (60 nm) were prepared using a Leica UC7, mounted on copper electron microscopy grids and counter stained using Uranyless (Science services) and Reynolds lead citrate. The sections were examined at varying nominal magnifications using a 120 kV FEI Tecnai Spirit TEM microscope equipped with a F416 CMOS camera (TVIPS).

### Morphology of class IV dendritic arborization neurons

Third instar male larvae were dissected following a ventral midline incision for daC neurons visualization. Transgenically expressed Membrane-GFP signal was enhanced using the primary antibodies: rat-anti-mCD8 (1/100, MCD0800, Invitrogen) and rabbit-anti-GFP (1/600, A11122, Invitrogen), secondary antibodies goat-anti-rat Alexa Fluor 488 (1/200; Thermo Fisher Scientific A-11006) and goat anti-rabbit Alexa Fluor 488 (1/250 Molecular Probes A11008) following the same procedure as described in NMJ immunohistochemistry section. Z-stack images were obtained per genotype with SP5 (Leica Microsystems) confocal microscope and imported into NeuronStudio (version 0.9.92, http://research.mssm.edu/cnic/tools-ns.html) for generation of neuronal tracings and Sholl analysis (10μm interval) [124]. Dendritic tracings were analyzed with L-Measure (version v5.2 [125] and statistical significance was analyzed using the One-Sample T-Test in GraphPad Prism. Graphical dendritic reconstructions were obtained with Neromantic [126].

### Light-off jump habituation assay

Light-off jump habituation was performed as previously described with a few modifications [127]. Briefly, three to six days old male flies were tested. Flies were transferred into individual chambers of two 16-unit habituation systems (Aktogen ltd., Hungary) and, after 5 min adaptation; they were exposed to 100 light-off pulses (15 ms each) with 1-second interval between the pulses. Jump responses were recorded as accompanying wing vibration noise with two sensitive microphones placed in each chamber. Data were collected and analyzed by a custom Labview Software (National Instruments). Only genotypes with ≥ 50% of flies responding with a jump to at least one of the first five light-off pulses were analyzed for habituation. Flies were considered habituated when they stopped jumping for five consecutive light-off pulses (no-jump criterion). Habituation was scored as the number of trials required to reach the no-jump criterion (trials to criterion, TTC).

### Social space assay

The social space assay was performed as previously detailed [128]. Flies were acclimatized to the behavioral room (25°C, 50% humidity) for two hours prior to being added via aspiration to the assay chamber, a 2D-like vertical chamber of triangular shape, (dimensions: 16.5 cm by 14.5 cm by 5.96 cm). All flies were tested on 3 independent days, in groups of 12 to17 flies. After 30 min, when flies had settled in their preferred locations, a picture of the chamber was taken, and distances between flies were analyzed with ImageJ (National Institutes of Health, Bethesda, Maryland, United States). Data representation and statistical analyses were performed using the statistical program GraphPad Prism (version 7.00 for Mac, GraphPad Software).

## Supporting information

S1 Fig GFP-FoxP is expressed in neurons but not in glia. Maximum projection of *w;;GFP-FoxP* brains co-immunostained with anti-GFP (green) and **(A)** anti-Elav (magenta), identifying neurons, **(B)** anti-Repo (magenta) labelling glia. Scale bar corresponds to 20 μm. Images were obtained from male brains at 0–2 hours post-eclosion.
(TIF)

S2 Fig GFP-FoxP is expressed in the larval brain and in the adult thoracic-abdominal ganglion. **(A)** Maximum projection of *w;;GFP-FoxP* flies larval brain from L3 wandering stage. **(B)** Maximum projection of thoracic-abdominal ganglion of *w;;GFP-FoxP* flies, GFP-FoxP (green), anti-Brp (magenta), scale bar corresponds to 50μm. Images were obtained from male brains at 0–2 hours post-eclosion.
(TIF)

S3 Fig FoxP is not expressed in octopaminergic neurons and expressed in one specific dopaminergic neuron. **(A)** Maximum projection of brain image stacks. **(A)** *w; Tdc2-*GAL4*/ UAS-GFPnls* flies co-immunostained with anti-FoxP (magenta) anti-GFP (green). **(B)** Wildtype flies co-immunostained with anti-FoxP (magenta) anti-TH (green) **(B´)** Magnification of B highlighted with a yellow square in the original images. Scale bar 50 μm in A, B and 10 μm in B’. Images were obtained from male brains at 0–2 hours post-eclosion.
(TIF)

S4 Fig Panneuronal *FoxP* downregulation leads to reduced fitness. **(A)** Locomotion trajectories of representative flies of indicated conditions. **(B)** Total distance (in cm) walked in 7 minutes of locomotion tracking. **(C)** *Drosophila* escape response, assessed in the island assay. Graphs show % of flies that remain on the platform over time (10 seconds). Data are represented as average and SEM of a minimum of 3 independent experiments per genotype. The genotypes depicted in the graphs are *w/Y; UAS-Dcr2/+; elav-*GAL4*/+* (control), *w/Y; UAS-Dcr2/+; elav-*GAL4/*UAS-FoxP-RNAi1* (*FoxP-RNAi1*), *w/Y; UAS-Dcr2/+; elav-*GAL4/*UAS-FoxP-RNAi2* (*FoxP-RNAi2*). One-way ANOVAs with Tukey’s multiple comparison test were used to compare each condition and determine significant differences (*p<0.05, **p<0.01 and ***p<0.001). For the underlying numerical data see S6 and S7 Tables.
(TIF)

S5 Fig *FoxP* levels determine MB α-lobe size. Maximum projection of MB image stacks of fly brains stained with anti-Fasll. Scale bar corresponds to 20 μm. *FoxP* panneuronal downregulation **(A)** *w/Y; UAS-Dcr2/+; elav-*GAL4*/+* (control), **(B)** *w/Y; UAS-Dcr2/+; elav-*GAL4/*UAS-FoxP-RNAi1* (*FoxP-RNAi1*), **(C)** *w/Y; UAS-Dcr2/+; elav-*GAL4/*UAS-FoxP-RNAi2* (*FoxP-RNAi2*). *FoxP* MB downregulation **(D)** *w*, *UAS-Dcr2/Y; 247-*GAL4*/+; +/+* (control), **(E)** *w*, *UAS-Dcr2/Y; 247-*GAL4*/+; UAS-FoxP-RNAi1*/*+* (*FoxP-RNAi1*) and **(F)** *w*, *UAS-Dcr2/Y; 247*-GAL*/+; UAS-FoxP-RNAi2/+* (*FoxP-RNAi2*). *FoxP* MB overexpression **(G)** *w/Y; 247-*GAL4*/+; elav*-GAL4/*+* (control), **(H)** *w/Y; 247-*GAL4*/+; elav*-GAL4*/ UAS*-*FoxP-I1* (*UAS*-*FoxP-I1)* and **(I)** *w/Y; 247-*GAL4*/+; elav*-GAL4/*UAS*-*FoxP-I2 (UAS*-*FoxP-I2)*. **(J-L)** MB α-lobe areas of the indicated genotypes. Scale bar corresponds to 20 μm. Data are represented as average and SEM of a minimum of 11 α-lobes. T-test between conditions was performed to determine significance (***, p<0.001). Images were obtained from male brains at 0–2 hours post-eclosion. For the underlying numerical data see S8 Table.
(TIF)

S6 Fig *FoxP* knockdown by RNAi leads to an expanded Dlg1-labelled synaptic area, phenocopying defects of *FoxP*^*-/-*^ mutants. Muscle four type 1b NMJs of wandering L3 larvae. Dlg1 immunostainings of male larva with following genotypes: **(A)** *w/Y; UAS-Dcr2/+; elav-*GAL4*/+*, **(B)** *w/Y; UAS-Dcr2/+; elav-*GAL4/*UAS-FoxP-RNAi1*, **(C)** *w/Y; UAS-Dcr2/+; elav-*GAL4/*UAS-FoxP-RNAi2*. **(D)** *w/Y; Mef2-*GAL4*/+; +/+*, **(E)** *w/Y; Mef2-*GAL4*/+; UAS-FoxP-RNAi1*/*+* and **(F)** *w/Y; Mef2*-GAL*/+; UAS-FoxP-RNAi2/+*. **(G, H)** Average Dlg1-labelled postsynaptic area of *FoxP RNAi1* and *RNAi2* downregulated with **(G)** *elav-*GAL4 and **(H)** *Mef2*-GAL4. Differences between the average NMJ area of controls are likely due to differences in genetic background between the driver lines. Genetic background is a known variable in the determination of NMJ area [122]. Data are represented as average and SEM of a minimum of 31 independent biological replicates. One-way ANOVA with Dunn’s multiple comparison test was used to compare each condition against the control and determine significant differences (* p<0.05, *** p<0.001). For underlying numerical data see S11 Table.
(TIF)

S7 Fig *FoxP* RNAi-mediated knockdown in type IV da neurons leads to a decrease in dendritic field area and dendritic length, recapitulating *FoxP*^*-/-*^ phenotypes. **(A-C)** Confocal projections of class IV da neurons within segment A3 of wandering third instar larvae, visualized with the class IV da-specific GFP expression (*477*-GAL4>*UAS-mCD8*::*GFP*). Reconstructions are represented in blue, superimposed on the traced neurons. Scale bar: 100μm. **(A’-C’)** Areas of high magnifications are highlighted in the original image, scale bar 20μm. The following genotypes are depicted in the panels **(A)** *w/Y; 477*-GAL4>*UAS-mCD8*::*GFP/+* (Control), **(B)** *w/Y; 477-*GAL4>*UAS-mCD8*::*GFP/+; UAS-FoxP-RNAi1/+ (FoxP-RNAi1)* **(C)** *w/Y; 477*-GAL4>*UAS-mCD8*::*GFP/+; UAS-FoxP-RNAi2/+ (FoxP-RNAi2)*. **(D, K)** Sholl analysis of accumulative dendritic length, the graph indicates the sum of dendritic length in concentric circles from the soma situated every 10μm. **(E, L)** Sholl analysis of cumulative number of branching points; the graph indicates the sum of branching points located in concentric circles from the soma situated every 10μm. **(F-J)** Quantitative analysis of dendritic trees, *FoxP-RNAi1* and *FoxP-RNAi2* manifest a decrease in **(F)** dendritic field area. **(G)** *FoxP-RNAi1* manifests a decrease in average branch length. **(H)** Cumulative branch length and **(I)** number of endings are not affected in any of the RNAi knockdowns. Control (n = 9), *FoxP-RNAi1* (n = 5) and *FoxP-RNAi2* (n = 5). **(J)** Dendritic endings density (number of endings in 100μm^2^) is increased in *FoxP-RNAi1*. *Control* (n = 16), *FoxP-RNAi1* (n = 10) and *FoxP-RNAi2* (n = 10). *FoxP-RNAi1* is depicted in dark blue, *FoxP-RNAi2* is depicted in light blue, controls are depicted in grey. Data are presented as average with SEM. One-way ANOVA Dunn’s multiple comparison tests were used to compare each condition against the control and determine significances (*** p<0.001). For the underlying numerical data see S13 and S15 Tables.
(TIF)

S8 Fig *FoxP-I1* overexpression in type IV da neurons does not induce significant differences in dendritic morphology. **(A-E)** Quantitative analysis of dendritic trees of *w/Y; 477*-GAL4>*UAS-mCD8*::*GFP/+; +/+* (controls) and *w/Y; 477*-GAL4>*UAS-mCD8<GFP/+; UAS-FoxP-I1/+ (UAS-FoxP-I1)*. *UAS-FoxP-I1* do not present significant differences in **(B)** dendritic field area, **(C)** average branch length, **(D)** cumulative branch length and **(E)** number of endings. Controls (n = 5), *UAS-FoxP*-*I1* (n = 5). **(F)** Dendritic endings density (number of endings in 100μm^2^) is unaffected in *UAS-FoxP*-*I1*. Controls (n = 10), *UAS-FoxP*-*I1* (n = 10). *UAS-FoxP-I1* is depicted in dark green versus controls in black. **(G)** Sholl analysis of cumulative dendritic length; the graph indicates the sum of dendritic length in concentric circles from the soma situated every 10μm. **(H)** Sholl analysis of cumulative number of branching points; the graph indicates the sum of branching points located in concentric circles from the soma situated every 10μm. Data are presented as average with SEM. T-tests between conditions were performed for each parameter to determine significance. For the underlying numerical data see S13 and S16 Tables.
(TIF)

S9 Fig Decrease of FoxP expression in *FoxP* panneuronal knockdown flies and transheterozygous FoxP hypomorphic flies. Maximum projection of brain hemisphere of adult flies, stained with anti-FoxP in **(A)** *w/Y;UAS-Dcr2; elav*-GAL4/+ (controls), (B) *w/Y;UAS-Dcr2/+; elav-*GAL4/*UAS-FoxP-RNAi1* and **(C)** *w/Y;UAS-Dcr2/+; elav*-GAL4/*UAS-FoxP-RNAi2*. **(D)** wildtype (*Wt*) and **(E)** transheterozygous *FoxP* hypomorphic flies (*FoxP*^*-/5-SZ-3955*^). Scale bar: 100μm.
(TIF)

S1 Table Primer sequences for genomic PCR and Q-PCR. Primes 1–3: amplification of FoxP isoforms containing the gateway sequences for subsequent FoxP cloning. Primers 4–5: FoxP Tagging. Primers 6–8: amplification of FoxP isoforms. Primers 9–14: Q-PCR primer sequences to amplify cDNA corresponding to FoxP (common region), 1tub23cf and RNApopl2. Primers 15–16: Primer sequences to detect FoxP genomic region deleted in the FoxP mutants. Genomic or cDNA sequences are leveled in black, gateway sequence is labeled in red, tagging cassette is labeled in blue.
(XLSX)

S2 Table Raw Q-PCR data corresponding to Fig 2A and 2B. Data is depicted as relative *FoxP* expression. S 2.1Table: Fold change indicates the proportion between FoxP relative expression at the indicated stage with FoxP expression at embryonic stage (being the lowest relative expression level). S2.2 Table: Fold change indicates the proportion between FoxP relative expression in neural tissues and the rest of the body in each developmental stage.
(XLSX)

S3 Table Raw data corresponding to Fig 2C. Percentage of co-localization between FoxP labeling signal and anti-Elav or anti-Repo signal in wild type brain image-stacks.
(XLSX)

S4 Table Raw data corresponding to Fig 4A and 4C. Percentage of dead pupa per vial.
(XLSX)

S5 Table Raw data collected in the survival assay corresponding to Fig 4B–4D. Percentage of flies alive over days post-eclosion per vial.
(XLSX)

S6 Table Raw data collected in the locomotion assay corresponding to Figs 4G–4I and S4B. Distance is expressed in cm.
(XLSX)

S7 Table Raw data collected in the island assay corresponding to Figs 4H–4J and S4C. Percentage of flies remaining in the platform over time.
(XLSX)

S8 Table Raw data corresponding to Figs 5C and S5J–S5L. MB α-lobes area in μm^2^.
(XLSX)

S9 Table Raw data corresponding to Fig 6C, 6D, 6G and 6H. Quantitative measurements of FoxP mutants NMJ features with fluorescent microscopy.
(XLSX)

S10 Table Raw data corresponding to Fig 6O and 6P. Quantitative measurements of FoxP mutants NMJ features with electron microscopy.
(XLSX)

S11 Table Raw data corresponding to Fig 7G and 7H. Dlg1-labelled post-synaptic area in μm^2^.
(XLSX)

S12 Table *FoxP*-*I1* and *-I2* overexpression does not lead to synaptic defects in Dlg1-labelled NMJ. Quantitative measurements of six NMJ features of muscle four type 1b of wandering L3 larvae. The following genotypes where analyzed control (*w/Y;; elav-*GAL4*/+)*, *UAS-FoxP-I1* (*w/Y;; elav-*GAL4/*UAS-FoxP-I1)*, *UAS-FoxP-I1* (*w/Y;; elav-*GAL4/*UAS-FoxP-I2)*.
(XLSX)

S13 Table Raw data corresponding to dendritic arbor analyses Figs 7E–7N and S7F–S7J and S8A7E–S8E. (XLSX)

S14 Table Raw data corresponding to the Sholl analyses Fig 8A’–8C’). (XLSX)

S15 Table Raw data corresponding to the Sholl analyses S7D, S7E, S7K and S7L Fig. (XLSX)

S16 Table Raw data corresponding to the Sholl analyses S8F and S8G Fig. (XLSX)

S17 Table Raw data collected in the habituation assay corresponding to Fig 8A’–8C’. Number of trials required to reach the no-jump criterion (trials to criterion, TTC).
(XLSX)

S1 Text FoxP mutant’s deletion map. (DOCX)

S1 Video GFP expression in GFP-tagged FoxP line. Confocal image stack of a one day adult brain corresponding to *w;;GFP-FoxP* flies co-immunostained with anti-GFP (green).
(AVI)
