# Supplementary figures and images for: Conserved regulation of neurodevelopmental processes and behavior by FoxP in Drosophila
Source: PLoS One. 2019 Feb 12;14(2):e0211652. doi: 10.1371/journal.pone.0211652 (PMC6372147; doi:10.1371/journal.pone.0211652)

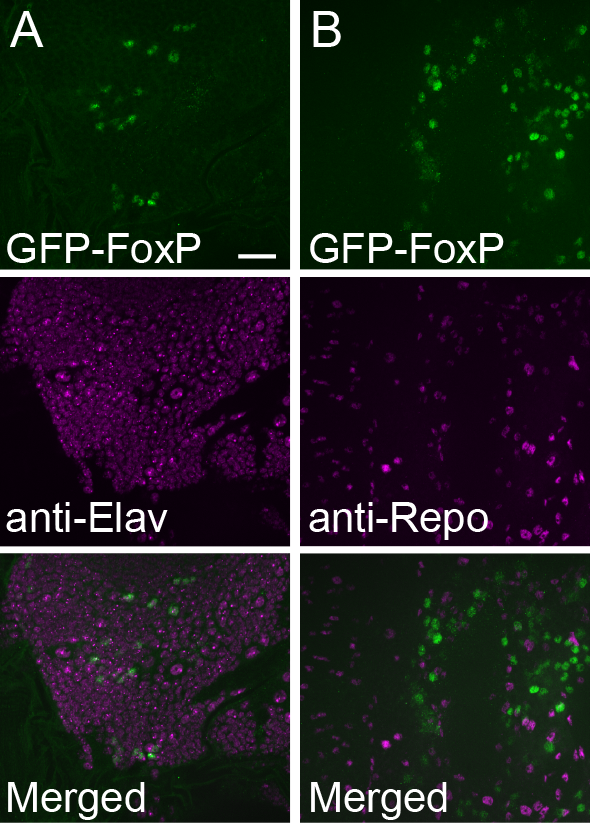

Supplement: S1 Fig — Maximum projection of w;;GFP-FoxP brains co-immunostained with anti-GFP (green) and (A) anti-Elav (magenta), identifying neurons, (B) anti-Repo (magenta) labelling glia. Scale bar corresponds to 20 μm. Images were obtained from male brains at 0–2 hours post-eclosion. (TIF) [file pone.0211652.s001.tif]

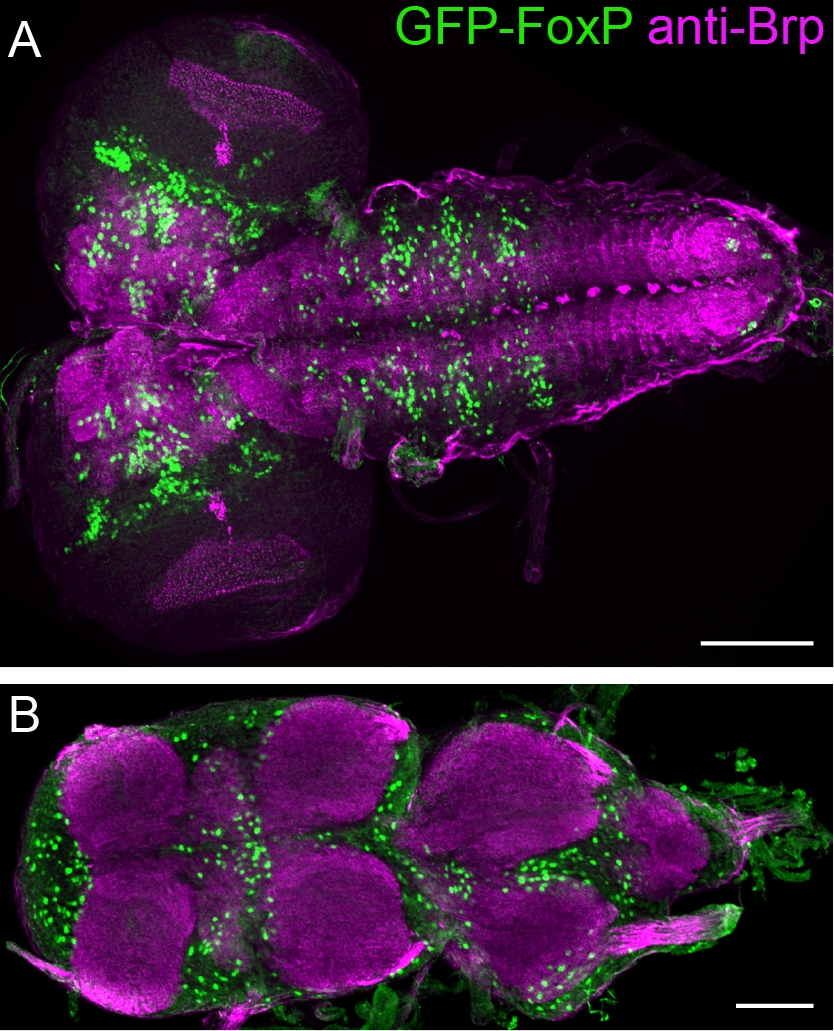

Supplement: S2 Fig — (A) Maximum projection of w;;GFP-FoxP flies larval brain from L3 wandering stage. (B) Maximum projection of thoracic-abdominal ganglion of w;;GFP-FoxP flies, GFP-FoxP (green), anti-Brp (magenta), scale bar corresponds to 50μm. Images were obtained from male brains at 0–2 hours post-eclosion. (TIF) [file pone.0211652.s002.tif]

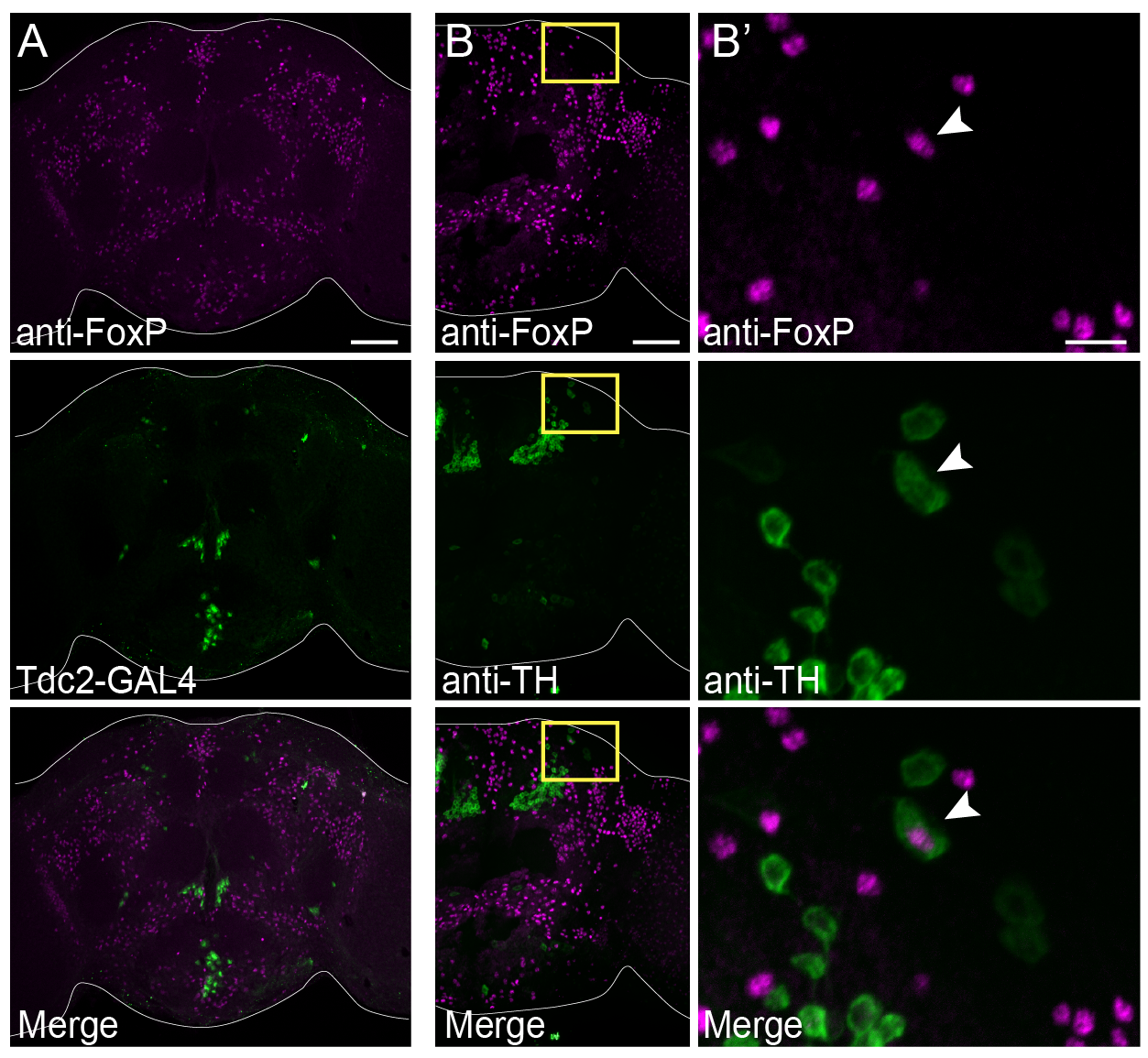

Supplement: S3 Fig — (A) Maximum projection of brain image stacks. (A) w; Tdc2-GAL4/ UAS-GFPnls flies co-immunostained with anti-FoxP (magenta) anti-GFP (green). (B) Wildtype flies co-immunostained with anti-FoxP (magenta) anti-TH (green) (B´) Magnification of B highlighted with a yellow square in the original images. Scale bar 50 μm in A, B and 10 μm in B’. Images were obtained from male brains at 0–2 hours post-eclosion. (TIF) [file pone.0211652.s003.tif]

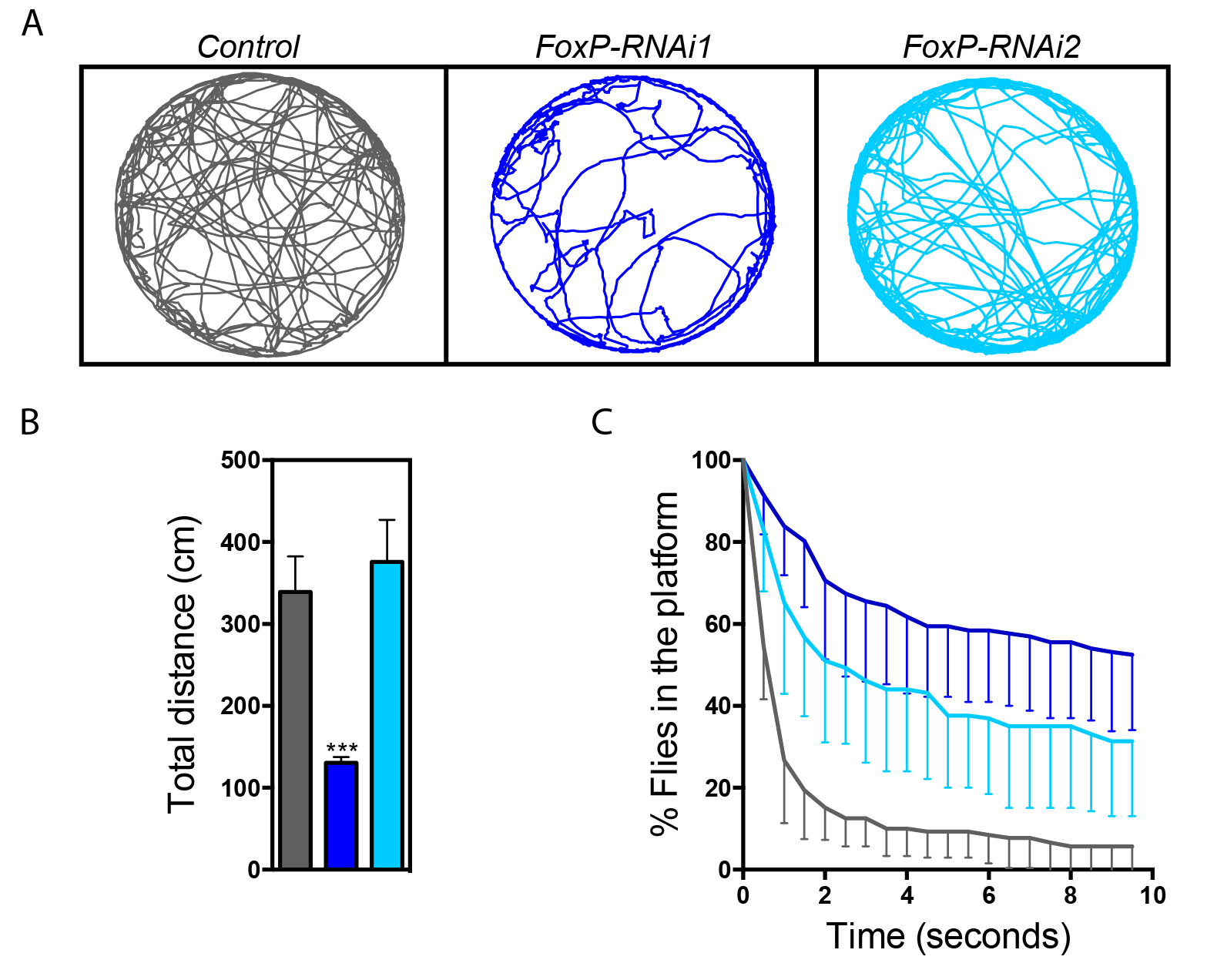

Supplement: S4 Fig — (A) Locomotion trajectories of representative flies of indicated conditions. (B) Total distance (in cm) walked in 7 minutes of locomotion tracking. (C) Drosophila escape response, assessed in the island assay. Graphs show % of flies that remain on the platform over time (10 seconds). Data are represented as average and SEM of a minimum of 3 independent experiments per genotype. The genotypes depicted in the graphs are w/Y; UAS-Dcr2/+; elav-GAL4/+ (control), w/Y; UAS-Dcr2/+; elav-GAL4/UAS-FoxP-RNAi1 (FoxP-RNAi1), w/Y; UAS-Dcr2/+; elav-GAL4/UAS-FoxP-RNAi2 (FoxP-RNAi2). One-way ANOVAs with Tukey’s multiple comparison test were used to compare each condition and determine significant differences (*p<0.05, **p<0.01 and ***p<0.001). For the underlying numerical data see S6 and S7 Tables. (TIF) [file pone.0211652.s004.tif]

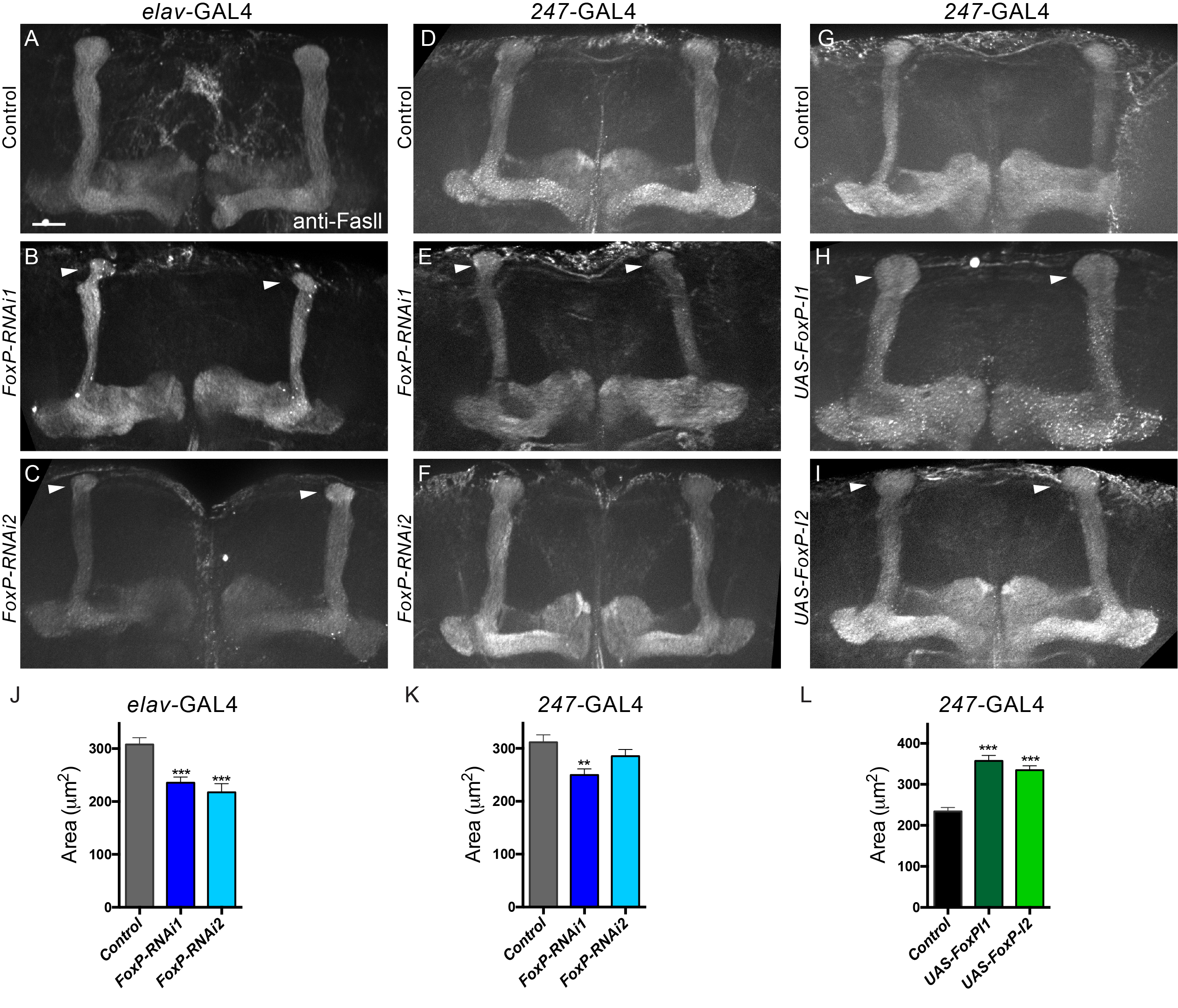

Supplement: S5 Fig — Maximum projection of MB image stacks of fly brains stained with anti-Fasll. Scale bar corresponds to 20 μm. FoxP panneuronal downregulation (A) w/Y; UAS-Dcr2/+; elav-GAL4/+ (control), (B) w/Y; UAS-Dcr2/+; elav-GAL4/UAS-FoxP-RNAi1 (FoxP-RNAi1), (C) w/Y; UAS-Dcr2/+; elav-GAL4/UAS-FoxP-RNAi2 (FoxP-RNAi2). FoxP MB downregulation (D) w, UAS-Dcr2/Y; 247-GAL4/+; +/+ (control), (E) w, UAS-Dcr2/Y; 247-GAL4/+; UAS-FoxP-RNAi1/+ (FoxP-RNAi1) and (F) w, UAS-Dcr2/Y; 247-GAL/+; UAS-FoxP-RNAi2/+ (FoxP-RNAi2). FoxP MB overexpression (G) w/Y; 247-GAL4/+; elav-GAL4/+ (control), (H) w/Y; 247-GAL4/+; elav-GAL4/ UAS-FoxP-I1 (UAS-FoxP-I1) and (I) w/Y; 247-GAL4/+; elav-GAL4/UAS-FoxP-I2 (UAS-FoxP-I2). (J-L) MB α-lobe areas of the indicated genotypes. Scale bar corresponds to 20 μm. Data are represented as average and SEM of a minimum of 11 α-lobes. T-test between conditions was performed to determine significance (***, p<0.001). Images were obtained from male brains at 0–2 hours post-eclosion. For the underlying numerical data see S8 Table. (TIF) [file pone.0211652.s005.tif]

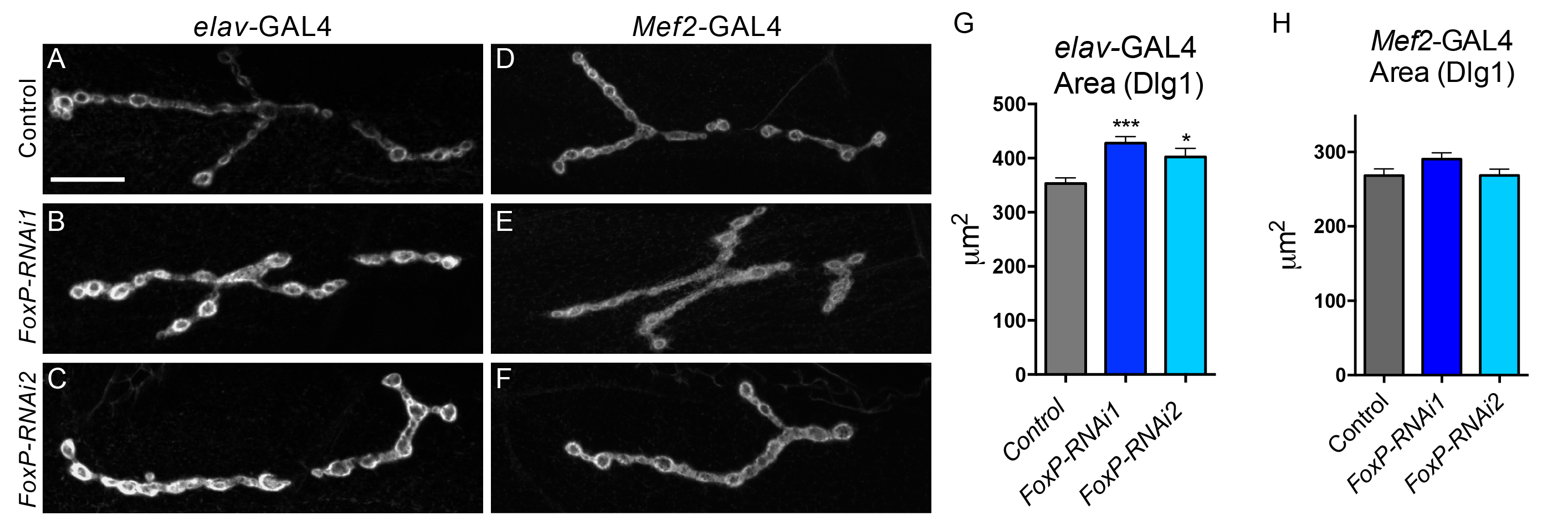

Supplement: S6 Fig — Muscle four type 1b NMJs of wandering L3 larvae. Dlg1 immunostainings of male larva with following genotypes: (A) w/Y; UAS-Dcr2/+; elav-GAL4/+, (B) w/Y; UAS-Dcr2/+; elav-GAL4/UAS-FoxP-RNAi1, (C) w/Y; UAS-Dcr2/+; elav-GAL4/UAS-FoxP-RNAi2. (D) w/Y; Mef2-GAL4/+; +/+, (E) w/Y; Mef2-GAL4/+; UAS-FoxP-RNAi1/+ and (F) w/Y; Mef2-GAL/+; UAS-FoxP-RNAi2/+. (G, H) Average Dlg1-labelled postsynaptic area of FoxP RNAi1 and RNAi2 downregulated with (G) elav-GAL4 and (H) Mef2-GAL4. Differences between the average NMJ area of controls are likely due to differences in genetic background between the driver lines. Genetic background is a known variable in the determination of NMJ area [122]. Data are represented as average and SEM of a minimum of 31 independent biological replicates. One-way ANOVA with Dunn’s multiple comparison test was used to compare each condition against the control and determine significant differences (* p<0.05, *** p<0.001). For underlying numerical data see S11 Table. (TIF) [file pone.0211652.s006.tif]

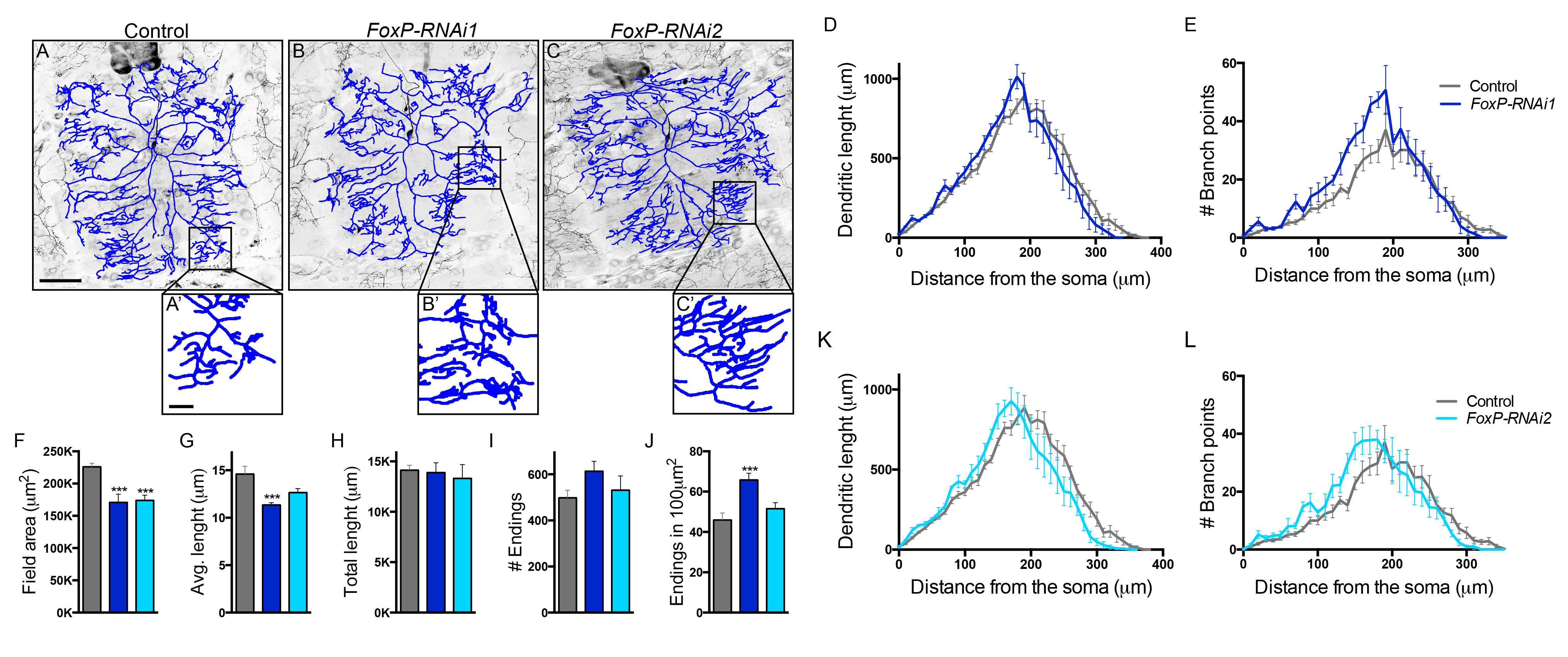

Supplement: S7 Fig — (A-C) Confocal projections of class IV da neurons within segment A3 of wandering third instar larvae, visualized with the class IV da-specific GFP expression (477-GAL4>UAS-mCD8::GFP). Reconstructions are represented in blue, superimposed on the traced neurons. Scale bar: 100μm. (A’-C’) Areas of high magnifications are highlighted in the original image, scale bar 20μm. The following genotypes are depicted in the panels (A) w/Y; 477-GAL4>UAS-mCD8::GFP/+ (Control), (B) w/Y; 477-GAL4>UAS-mCD8::GFP/+; UAS-FoxP-RNAi1/+ (FoxP-RNAi1) (C) w/Y; 477-GAL4>UAS-mCD8::GFP/+; UAS-FoxP-RNAi2/+ (FoxP-RNAi2). (D, K) Sholl analysis of accumulative dendritic length, the graph indicates the sum of dendritic length in concentric circles from the soma situated every 10μm. (E, L) Sholl analysis of cumulative number of branching points; the graph indicates the sum of branching points located in concentric circles from the soma situated every 10μm. (F-J) Quantitative analysis of dendritic trees, FoxP-RNAi1 and FoxP-RNAi2 manifest a decrease in (F) dendritic field area. (G) FoxP-RNAi1 manifests a decrease in average branch length. (H) Cumulative branch length and (I) number of endings are not affected in any of the RNAi knockdowns. Control (n = 9), FoxP-RNAi1 (n = 5) and FoxP-RNAi2 (n = 5). (J) Dendritic endings density (number of endings in 100μm2) is increased in FoxP-RNAi1. Control (n = 16), FoxP-RNAi1 (n = 10) and FoxP-RNAi2 (n = 10). FoxP-RNAi1 is depicted in dark blue, FoxP-RNAi2 is depicted in light blue, controls are depicted in grey. Data are presented as average with SEM. One-way ANOVA Dunn’s multiple comparison tests were used to compare each condition against the control and determine significances (*** p<0.001). For the underlying numerical data see S13 and S15 Tables. (TIF) [file pone.0211652.s007.tif]

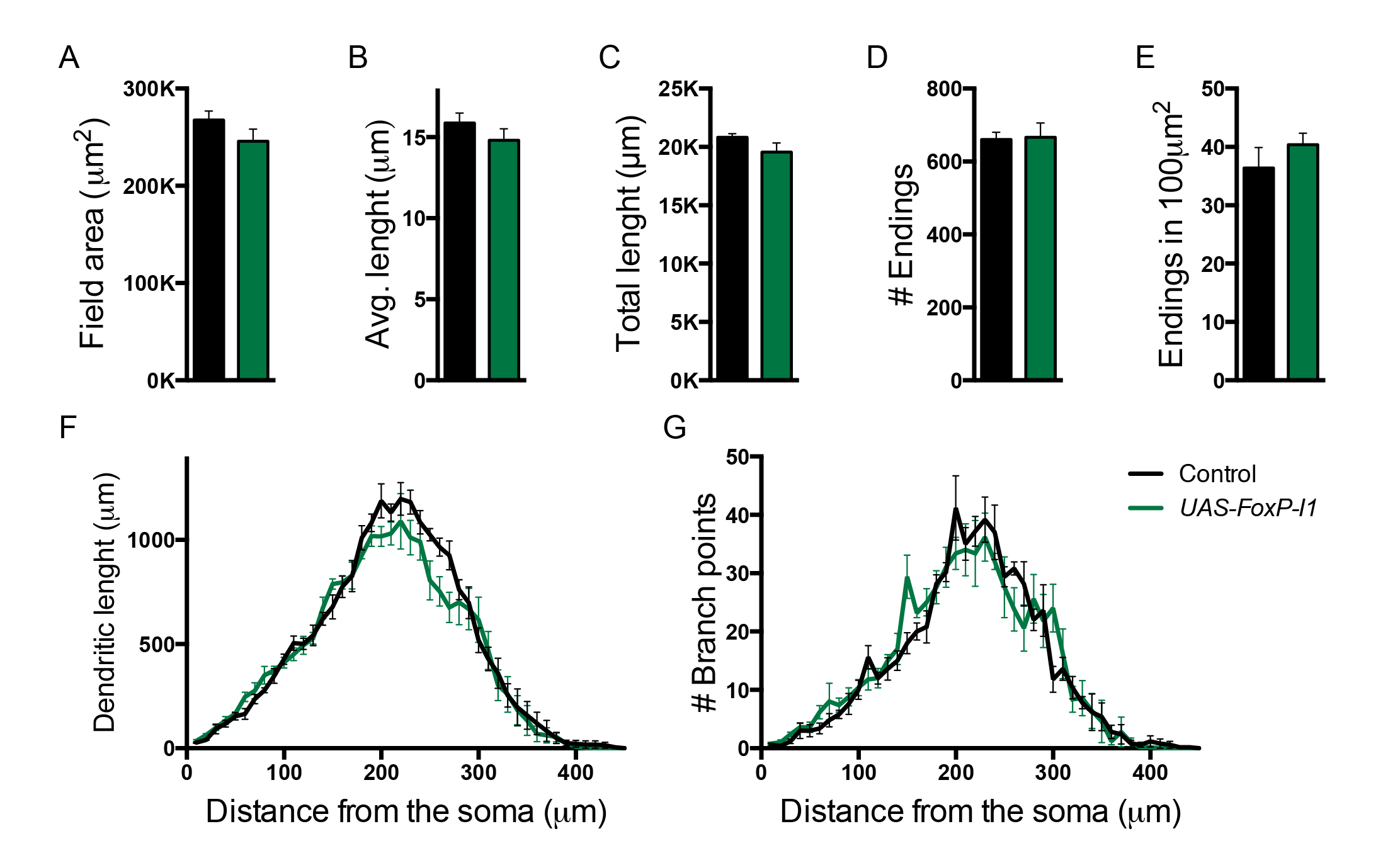

Supplement: S8 Fig — (A-E) Quantitative analysis of dendritic trees of w/Y; 477-GAL4>UAS-mCD8::GFP/+; +/+ (controls) and w/Y; 477-GAL4>UAS-mCD8<GFP/+; UAS-FoxP-I1/+ (UAS-FoxP-I1). UAS-FoxP-I1 do not present significant differences in (B) dendritic field area, (C) average branch length, (D) cumulative branch length and (E) number of endings. Controls (n = 5), UAS-FoxP-I1 (n = 5). (F) Dendritic endings density (number of endings in 100μm2) is unaffected in UAS-FoxP-I1. Controls (n = 10), UAS-FoxP-I1 (n = 10). UAS-FoxP-I1 is depicted in dark green versus controls in black. (G) Sholl analysis of cumulative dendritic length; the graph indicates the sum of dendritic length in concentric circles from the soma situated every 10μm. (H) Sholl analysis of cumulative number of branching points; the graph indicates the sum of branching points located in concentric circles from the soma situated every 10μm. Data are presented as average with SEM. T-tests between conditions were performed for each parameter to determine significance. For the underlying numerical data see S13 and S16 Tables. (TIF) [file pone.0211652.s008.tif]

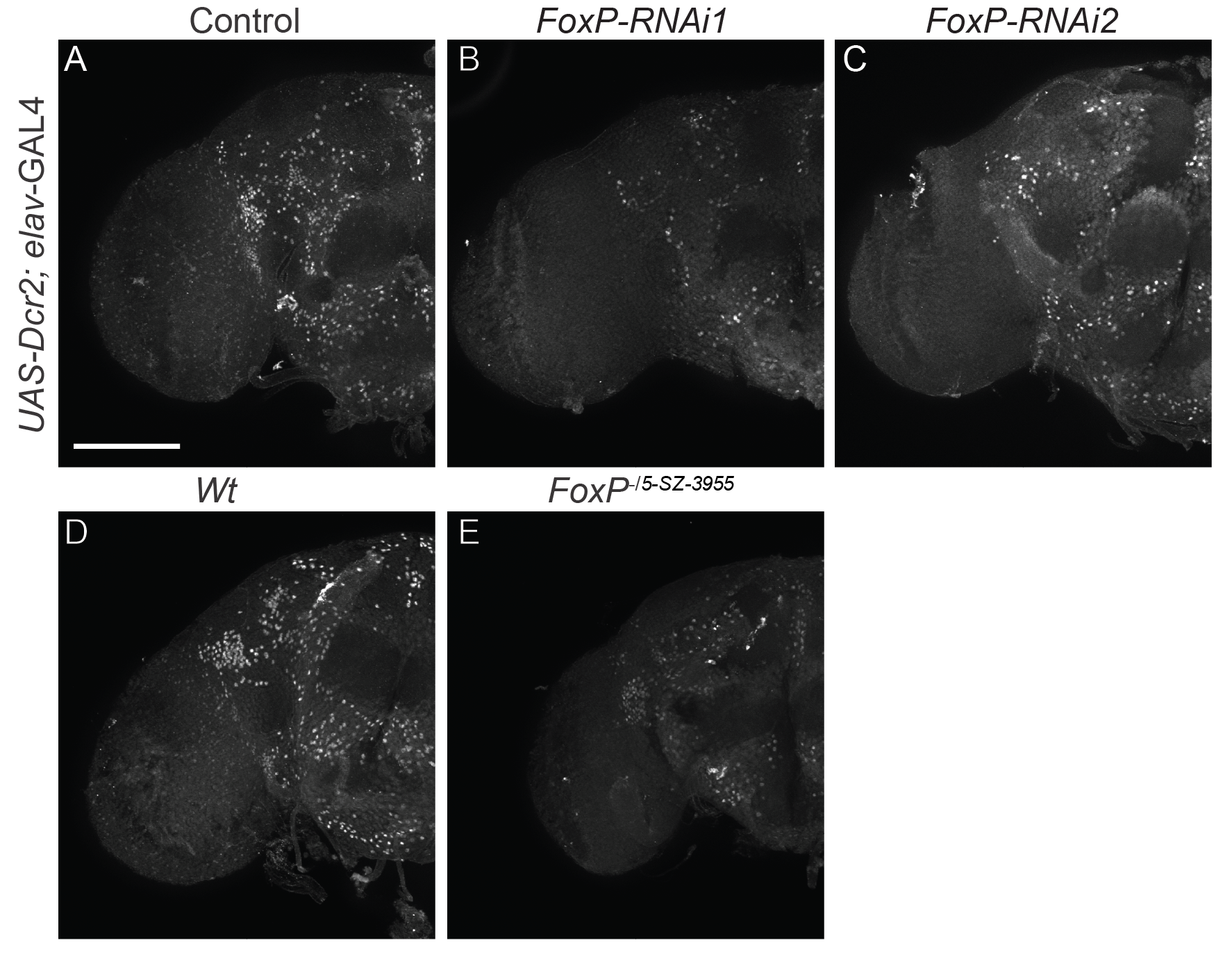

Supplement: S9 Fig — Maximum projection of brain hemisphere of adult flies, stained with anti-FoxP in (A) w/Y;UAS-Dcr2; elav-GAL4/+ (controls), (B) w/Y;UAS-Dcr2/+; elav-GAL4/UAS-FoxP-RNAi1 and (C) w/Y;UAS-Dcr2/+; elav-GAL4/UAS-FoxP-RNAi2. (D) wildtype (Wt) and (E) transheterozygous FoxP hypomorphic flies (FoxP-/5-SZ-3955). Scale bar: 100μm. (TIF) [file pone.0211652.s009.tif]
