# Supplementary material for: Conserved regulation of neurodevelopmental processes and behavior by FoxP in Drosophila
Source: PLoS One. 2019 Feb 12;14(2):e0211652. doi: 10.1371/journal.pone.0211652 (PMC6372147; doi:10.1371/journal.pone.0211652)
Supplement: S1 Text — (DOCX) [file pone.0211652.s027.docx]

**FoxP deletion map:**

Deletion 4330 bp Insertion 2bp GA

*FoxP* Coding region

*hyd coding region*

*alfa-Tubulin* coding region

introns

EXONS

UTR

~~deletion~~

insertion

5´

caagcttctttcgagaagcttataatataaacatttcaatagtttgctcagcacaaatag

ttaaatatgaacaaattaatcagcatgtatgcacatgcactcacacatgctaccgttcac

atgtcgcacacattcgcacacacacacatatgcgaatggttagagaattaattgtggatg

aaaagtgggaaacgcaagtgcatttcgcaaattctgcggctaatgtttacagaaccgcgt

ggacggcactagatgaggtaagtccgccagtgtctgtggctcgcgttttacgttaatgat

catatgtacatatcttcctgcgtatacatatagctgcgtacctacatattttcacataac

aaatacatgcagccgtacgtacacaattacacggaattccgcgttcacatgtgcgtaatt

gcggtacgcgaggattatatttacCTTTCAATAAACTGATCGTCCGAGCCCGGTAGCGGT

TGCAAAACAAATTGCATGGAAACCATTTTAATTTTTCAACTATTCATGTACACAGTGTCC

TCGTTGAATTTTCATGTCACAAAACGTTGAGAGACTTTCAGAGTGACCGTACtggctaga

gctgggacgaaaaccgatgccgcccgactgtatcgatggttgccggaaaattcgacggta

tcgatacattctccaaacaaatttattggtaaacaaaaactttactttcaaattaaattg

acattgactacaatatgtgtacttaggctgtatatattttattaaatttaagatacagaa

gttcacgaatgcgtccattcttatcggttgtttttccttaaatagtggcagtccaaaaaa

tctccaatagaatatactaaaatatactaaagaccgctttaaaataatgaattggtagaa

aactttgattgacaaacttttgagaagccgatttaaccgcttttacgtcgcgtaaaccgt

ttgcttaggaactagaaataaaattcagtttgttcactttGTTGTATATTTGAGGAATTT

ATTTAGGTCACTGAGTATCGTTAATGATCTTAAATTAGAAGGAAATTAGATAAGTCAGCA

GAAAAGCATATACGTTTACTAAGGAGTTTCCAAATCTAAAGCTCTCAAGGGTCCAGATTT

TTAACAGAAGGTATACCTGAAAATAGTTTCCTTAAAAAAAAATCATATTGTGACTTTTAA

AACAGTTTTGTTAAAATTTTATCAAGCTATAGCTTTTAAATGTTTTCTCCCTTGCTGAAA

AGTGAAAATATTCATATATGTTGTACTCGTATATTAAAATCGTGTTATAAGTGGAGTTAT

TAAAGCAAGTTTAAACACATTAATTTTGCTTTAATAATTTAACGTAGTTCTTTCGTTTAT

TTTATAGCATTAGTGAAATTTATTTTAAAAGTATTTTTTCATTTCTATTTGGGAAATTAT

ACATAGAACCATGTGTATGTACAGATATAATTTCCGAATCCGAACCTTCTTACCAAACGC

AGATTACTCGACAATGCATCTAAATATATGTATAGTTAATGATATATTATTTATCGATTG

TGCTCATTGGCACTACTCTCGTACATATTAGTAGAATATTCATCGATTGTCAAGTGGCTG

TTACTTTCGATCATACCCGAGTCCATATTGCTATAGTCCTTGGACGCTTTACTTAAACCA

TGA~~CACTACCGCAATAGGGAAGACAACCAATTCTGCCTAAATCATTTGGATTATTGGAATCT~~

~~AGAGGATTATCAGCACCCGGTGGCAAACTAGTGCAATGTTGCGTACAATTGTCGCAGGGA~~

~~TTCTTATCAGTGGGCACACCATTGCCGGATTGGCATGAATTTGGGGAGGAGGACGGTTCA~~

~~TATTTCCGGGGTCTCCCTCTCGACAAGTGCCGCCTTTTGACAAACTCATTATCGTCGACC~~

~~ATCCAAAACGAGCCAAAGTCATCTTCATAACGTACAAAGCACTTGTGTAAGGAAAGGTTC~~

~~GTACGAATCGCATTctttatgattggttggacaaaacaagagccccatcaagagtcagaa~~

~~acaggaaattgcattatcgtgaaaggaagaaaaagttcattgagatattaaggttagttc~~

~~atttgtcttcggtcttcttattttcatttttCATAACACGCGAAATCCTGGATAATCTTC~~

~~ATCAGCTAAATTCCAGACAGACAGACAGACAGATCGATGAGCAGACGGACATCATTTTGG~~

~~GTTTCTACCACTAAGAGATTAGAATCACTGTTTACAATTTGCGCTGTAAAAAGATCGTAT~~

~~AATGTCATATTATATCATTTCGATATAGGAGCATGCATGCAATGGCATATGTATATTTAC~~

~~TAACAGAGCAAAACGGAAGGAGTTTGGAGCAAAGTGAGTAGAATTAAACTATTTGAGACC~~

~~CACATACCCAATGTTCATGGCTACAAAATAGTTAGTGTCCGGCGAATTGGTAGCACCGGT~~

~~TAGATTGTTACCAATGCCAGCAGTGCGCTGCGGTCGTCTTTTGTAAAACTCAATCTCATC~~

~~GACAGTCCAAACTGCTCCTTTCACATTCTCAACCCGCATAAAGCACTTATGAAGGGACAG~~

~~ATTGTGCCGGACTGCGTTctaatgatatatattatcagttagcataggccaaacttaatt~~

~~tgattattttaagacatataaaaaggaaatggaataaaacaggatctgacctgtgtagct~~

~~taccttgttatatatcacttgtatttagctctattatactcttaaatattggcaaagtag~~

~~tggacaatgtggaaaaggtagtatatagtatatatacagcggacaagctaggaacgccaa~~

~~tcggacaaacgagagttatacggacggacaaatggtcatatcagatcatagatatcatat~~

~~catatagcataaatactcaaattaaagttatgttgcataatatatagataatatggagga~~

~~aagaagatttacatatatggggcattttacCTTCCACGTAGCTGCGTTGCGCCGGAAGTA~~

~~GCAAAATGTGTTTTGGAACCAGTTGTAGATTTCGTTTAGGGTTAACTGCTTGTCAGGGGA~~

~~GTCAATTATAGCctataaataaaatatatataaaaaaaataaattaatgttatattagga~~

~~aattggtttttaaaattacCTGTCTTATGAGGGAAGCATAAGTAAAAGGCGGTCGTACAT~~

~~CAGCATTCTTATAAAACTCTCTGTTTCGATGGATTTctgcaaatttaaattcttattata~~

~~tgtcttccagaagcacaagacgaatagtcatcaagacgatcagtctacataatttactta~~

~~cCCTGTTGCACATCAAGACCTGCTCTTTCAAGCATATAGGGTAATCctatatccaaaaac~~

~~cattattatttttatacagtaatagtaaatttttcccaaacttacCCCCATTTATGGAAA~~

~~ATGTATTTTTGTCGTGGTTTCTCTTTTTGATCGAGCACAAGTTGGTGGAATTAACCATTG~~

~~GAAGATTCAGAGGACTCGGTGAATTTGTTTGTCGGATGGGTCGGCCTATGCTATTTACGG~~

~~TAAGCGGACTCCGGCAAAACTTTCCCTCTCTTCCGGGCACGTCcttatatttataacttt~~

~~tcattataccaatacttactcacataaaaagggacttacCTTCCTGTCGATTTTGGTGGG~~

~~AGACAAAAGCTGCTTGGACAAATACAAGTGATGCATCATTGCCTGCAAGCGATCTCGTTC~~

~~TTTTTGCAGGTGGGATTCCAGCTGGGAGACAACTTGCATTTGAACCCGTGCCTGTGCTGT~~

~~TGATCGATCGTCCAAACCATGTTCCGTATTCAGATGCctagttagataacataccctata~~

~~taattatttcatacctctaattgtactccacctacTTGACAAACGATGTGATGTCTTCCA~~

~~AATCCATTTCGCAACCAGGCCAACGGCATATGCCATGAGCAAATAACGGATGATAGTACT~~

~~TCCGCATGGCGAACTCATCGTGCATATAGCTGAAATCATTGTTGCACACCTCATTATCCT~~

~~TCGACCGCAAGAATCTCTCTGCATCGGAGAACATGAGCTTCTCCTGCTCGGATATGAACT~~

~~CGGGCACATTGTAGAAACCCAGATCGGGAACGGGAGCCATCATATGCTGACCCTGTATAT~~

~~GCATGTGTGGATTGGATTCGTGCTGCTGGGCCGCCGTTGAGGATTCATCGATGCTCGAGG~~

~~CCGGTGAGCTGCACTTAACTACATGTGAGGCAAAGGTGATCGAGTTATTGAGAAAGCCAG~~

~~TCGGCGGAAAACAATTGTTCTTAACAGCATCTGAGCATATGTCTGGTATGGAAATTTGGT~~

~~GTCTCGACTTCACTGATATCTCCTTCTGAATCGAACTCTTAAAGTCCGATTCCTTGGCAT~~

~~CCTCGGAATACTCGTCGTCATGTATCCGATGCATGTTTTTAAAACTGAAATTTATAATCA~~

~~TTACCATTGATCCTTTACCCAAGAGATGGGCATATGCAAATACACAGAAATAATCTCTTT~~

~~TTCGTCTGTCATTAGGAGTCATTTCAATAAAACTATCTTTACTCATATGCCGCCATTCCT~~

~~GTAAAACTCCGAATCAGAGTGATAGTTTAAGGATTTCTATTTATTACGCCCTATTTAATT~~

~~TCAGCTTAATTTTCCCGCACTATCATTGAGATGATACTTTCCCCAAAAGTTATGAATATT~~

~~TAACAACTTGGCGCATATAAGATTTAACCCCTACTAAAGGCCCAGTGCTAAATGAATTCA~~

~~GTGTAAATTAAATTAAACATTCTTTAAATGTTTGATTTAATTTTAAATATGTTACAAATA~~

~~AAATGCCACGTCATCCGGTGCAGTTAATCTGTAAATATAAAGGTTATTCATAGAAAGCAT~~

~~TTATCATATTTAGGCGTATAACAAGTTTAAGATCTCACAAGGGGATTGCATAGGTGGCAA~~

~~GGAAACCAGACACATCTTATTTTGCTAAAAAGAAACATATACAACATACATAACATAAGA~~

~~AACATACAGACGGAACGGTCGGACAAACGGAAGGACATACAAACGGACGGACAAACGGAC~~

~~GGACAAACGGACGGACAAACGGACGGACAAACGGACGGACAAACGGACGGACAAACGGAC~~

~~ATACAAACGGAAGGACATACAAACGGACGGACAAACGGATATAAAAACGGACGGACGTTT~~

~~TGGCTATAGGATGAACTTAAATGGAAGAGTATATGAGTTTTGCAGATAGGACCATCTTAC~~

~~GATTGTTTTAATCAAATTCCTGTAATGAAAAATTATATAGCCACCTAAAATAAGAATAAA~~

~~TGTAAATGGTGAAATATGTAAGTATTAAATGTTTTATAGGAAAAGAAGAAAGATATAAAA~~

~~GAGAAGAGAGAGGTATGGTTTTCATCACATTCGAAGATCCTGAAAAAATAGTATTATATT~~

~~CGATATACATAAGCTTTTTGTGTAAAGGCGAATAAAGATAAATATATTGTTGGTATTGTG~~

~~TAGATTTGCATTGTCTGTATTCGTTGGGGGCATTGATTATTCCAAGGGGTATATATGGGT~~

~~AAATAAAATATTCATATTTCATATGTATACATATCATACAATTTCTGTATAAGAACTGTT~~

~~TAAACTGGGACTTTTAAGATAAAGTTGTCTGCCTAGACTACTCAACAATATCTTAATTAA~~

~~CTTAACAGACTAGACTTAAAttttgcgaatacatacatgcacatacaattttgcagacaa~~

~~aagaggatacaattttgcatgtacatttacaattaaataacattcacagaggtttccaaa~~

~~gaacaatattatatttaataacgggcgaattttttaatacaacttttcacatatatgtaa~~

~~aattaatttaaacttaaaaactatcttaagaatatttttcgctgctactcctgaaaaggt~~

~~acgtctacgactactgaatttgctagaaatatgtgtacacatttttgcgcatgcgtttca~~

~~aaatgcatcctaaggctccattacacgtaacgatttcaaaaattaaaaacttttaaaagt~~

~~caacattaatt~~tgcccaattaatattaatatttaaagcaataagaaagtgataagtaggg

ttttttacggaggggtaatttccaataaatatatgatttaccaatttccttatatgagaa

agttttattcttattaacacaaaagttaaattccatctaaagacgaatatttcccattta

aataagccattttaagcaattGTGTTAAACTGTTTTATTGCGAAACTGTTTTATGCATTG

GTATTTGGTAGAAAGAAAGTGATGTTTATAAAGTTGTAATGGCTGTATCATTTGTTGACA

ACGTTTTCTCTAGTATTCCTCATCTTCCCCCAGTTCGGTGGTGGAGTCGATGCCTACCTC

CTCGTAGTCCTTCTCGAGGGCAGCGAGATCCTCGCGAGCCTCGGCGAACTCGCCCTCCTC

CATG 3’
